# Measuring the prediction difficulty of individual cases in a dataset using machine learning

**DOI:** 10.1038/s41598-024-61284-z

**Published:** 2024-05-07

**Authors:** Hyunjin Kwon, Matthew Greenberg, Colin Bruce Josephson, Joon Lee

**Affiliations:** 1 Department of Biomedical Engineering, Schulich School of Engineering, University of Calgary, Calgary, Alberta Canada; 2 Data Intelligence for Health Lab, Cumming School of Medicine, University of Calgary, Alberta, Canada; 3 Department of Mathematics and Statistics, Faculty of Science, University of Calgary, Calgary, Alberta Canada; 4 Department of Clinical Neurosciences, Cumming School of Medicine, University of Calgary, Calgary, Alberta Canada; 5 Department of Cardiac Sciences, Cumming School of Medicine, University of Calgary, Calgary, Alberta Canada; 6 Department of Community Health Sciences, Cumming School of Medicine, University of Calgary, Calgary, Alberta Canada; 7 Department of Preventive Medicine, School of Medicine, Kyung Hee University, Seoul, South Korea

**Keywords:** Computer science, Information technology

## Abstract

Different levels of prediction difficulty are one of the key factors that researchers encounter when applying machine learning to data. Although previous studies have introduced various metrics for assessing the prediction difficulty of individual cases, these metrics require specific dataset preconditions. In this paper, we propose three novel metrics for measuring the prediction difficulty of individual cases using fully-connected feedforward neural networks. The first metric is based on the complexity of the neural network needed to make a correct prediction. The second metric employs a pair of neural networks: one makes a prediction for a given case, and the other predicts whether the prediction made by the first model is likely to be correct. The third metric assesses the variability of the neural network’s predictions. We investigated these metrics using a variety of datasets, visualized their values, and compared them to fifteen existing metrics from the literature. The results demonstrate that the proposed case difficulty metrics were better able to differentiate various levels of difficulty than most of the existing metrics and show constant effectiveness across diverse datasets. We expect our metrics will provide researchers with a new perspective on understanding their datasets and applying machine learning in various fields.

## Introduction

In recent years, machine learning (ML) techniques have experienced significant growth and have been widely adopted in many fields. The expectation that ML has the potential to solve real-life problems such as disease prediction, product recommendation, and image recognition led to the development of ML applications in various fields^1^. Along with these developments, researchers are examining what deficiencies exist in the process of applying ML to data^2^. While there are many important factors, this paper focuses on the varying levels of prediction difficulty of individual cases in a dataset. Difficult cases are those that are hard to make correct predictions for^3^. For instance, in the same dataset, some cases may be easily classified by simple ML models, while some cases may require additional data and expertise to be classified correctly. Despite these differences in difficulty, most ML research does not consider such differences.

Previous studies found that prediction difficulty is one of the aspects inherent to an individual case^4–6^. Prediction difficulty is sometimes referred to as instance hardness which quantifies the likelihood of an instance being misclassified^4^. For intuitive understanding of the term, this paper uses the term ‘case difficulty’ to refer to instance hardness. The previous study proposed several metrics to measure case difficulty and found spatial overlap between target classes in a feature space was one of the principal contributors to misclassification^4^. Another study introduced a set of case difficulty metrics that can be obtained for an entire data set^5^. Yu and colleagues proposed a novel case difficulty metric defined as the proportion of a set of classifiers that make incorrect predictions^6^. The existing case difficulty metrics in the literature are listed in Table 1.

**Table 1:** Existing case difficulty metrics.

Method	Measure	Description
Neighborhood based method	k-Disagreeing Neighbors (kDN)^4^	Percentage of the k nearest neighbors of a case that do not belong to the same class
Decision tree-based method	Disjunct Class Percentage (DCP)^4^	Percentage of the cases in its disjunct, discovered rules in decision trees or rule-based learning algorithms, that belong to the same class
	Tree Depth (TD)^4^	The depth of the leaf node for a case in an induced decision tree. There are two ways to use the metrics, using pruned (TD_P) and unpruned (TD_U) decision trees
Naïve bayes-based method	Class Likelihood (CL)^4^	Likelihood of a case belonging to its class
	Class Likelihood Difference (CLD)^4^	The difference between the class likelihood of a case and the maximum likelihood for all the other classes
Class skew-based method	Minority Value (MV)^4^	The ratio of the number of cases that belong to the same class to the number of cases in the majority class
	Class Balance (CB)^4^	The ratio of the number of cases that belong to the same class to the number of cases in the dataset
Distance-based method	Fraction of nearby instances of different classes (N1)^5^	The percentage of cases of different classes connected to the minimum spanning tree
	Ratio of Intra/Extra Class Nearest Neighbor Distance (N2)^5^	The ratio of the distances between each example and its closest same class neighbor and its closest neighbor from another class
	Local Set Cardinality (LSC)^5^	The relative cardinality of the local set which is the number of the same class data points before reaching the nearest different class
	Local Set Radius (LSR)^5^	The normalized radius of the local set which is the number of the same class data points before reaching the nearest different class
	Harmfulness^5^	The number of cases having a case as their nearest enemy
	Usefulness^5^	The fraction of cases having a case in their local sets
Feature-based method	Fraction of features in overlapping areas (F1)^5^	The percentage of features of a case whose values lie in an overlapping region

The metrics in Table 1 can be grouped by the concepts that have been used to develop the metrics: Neighborhood-based method (kDN), decision tree-based methods (DCP, TD_P, TD_U), Naïve Bayes-based methods (CL, CLD), class skewness-based methods (MV, CB), and distance-based methods (N1, N2, LSC, LSR, Harmfulness, Usefulness), and feature-based method (F1).

However, the existing metrics have limitations, particularly in terms of their applicability to various types of datasets^7^. For example, the neighborhood-based method is vulnerable to datasets with categorical data, since distances cannot be clearly defined with respect to either nominal or ordinal categorical variables. The decision tree-based methods can show substantially different performances depending on the complexity of the dataset. Decision tree-based metrics can detect non-linear relationships through deeper trees. However, when the tree becomes excessively deep and attempts to create overly specific splits, it can lead to overfitting and poor performance on unseen data^8^. The Naïve Bayes-based methods work under the assumption that features in a dataset are independent, but features are often dependent on one another in real-world data^9^. The class skew-based methods are improper to use with balanced data since these metrics require a certain degree of class imbalance to calculate case difficulty. The distance-based methods cannot accurately calculate a distance in mixed data since the distance calculation involving both continuous and categorical variables is unclear. The Feature-based methods calculate case difficulty based on the overlap area between classes and are significantly affected by the presence and size of the overlap. Consequently, the existing metrics require several dataset preconditions to calculate case difficulty properly. However, it is often challenging to evaluate these preconditions of real-world datasets due to their diversity and complexity. Thus, there is a need for new universal metrics that are able to calculate case difficulty across various types of datasets.

In this paper, we propose novel metrics to measure case difficulty using fully-connected feedforward neural networks (NNs). There are two reasons why NNs are chosen over other ML models for developing metrics. First, NNs can act as universal approximators. According to the universal approximation theorem, a NN with a single hidden layer containing a sufficient number of neurons is capable of approximating any nonlinear continuous function^10^. Second, measuring model complexity in NNs is straightforward. The number of neurons represents the model's complexity, and this complexity can be used to calculate the difficulty of individual cases. Therefore, the NN is a suitable model for developing new metrics to measure case difficulty, which can perform equally well across datasets from different domains. We employ simulated and real-world data from various domains to develop these metrics. Simulated data allows for the visualization of the difficulty levels quantified by the metrics, whereas real-world datasets from diverse domains can evaluate the application of the proposed metrics in various fields. We evaluate our novel metrics through visualization and comparisons with the existing metrics. For simplicity, we focus only on classification problems based on tabular data.

In summary, case difficulty, which refers to the prediction difficulty of individual cases, is an inherent aspect of each case within a dataset. Previous studies have proposed several metrics to measure case difficulty. However, these existing metrics have shown limitations in their applicability across various datasets with specific data conditions. The conditions under which the existing metrics show weaknesses vary for each metric, but may include datasets with categorical variables, dependent features, balanced classes, and mixed continuous and categorical features or highly complex datasets. To address these limitations, we propose three novel case difficulty metrics, designed as universal tools for assessing case difficulty in any dataset.

## Methods

### Datasets

We used simulated datasets and real-world datasets to evaluate our case difficulty metrics. The simulated datasets were designed to have diverse shapes, including isotropic Gaussian blobs, interleaving crescent moons, and a large circle containing smaller circles. These datasets contained varying amounts of class overlap. The isotropic Gaussian blob data were generated using the make_blobs function from the sklearn.datasets in Python^11^. The parameters adjusting the standard deviation of the clusters were set to 2, 4, and 6, respectively, based on the desired levels of overlap. For the interleaving crescent moons data, a custom function named moon_shape was developed to generate clusters with controlled noise levels and parameters. The amount of overlap was manipulated by the parameters for the standard deviation of the Gaussian noise, and set to 0.1, 0.2, and 0.4, respectively. Data with a large circle containing smaller circles were created using the make_circles function from sklearn.datasets^11^. The scale factors between the inner and outer circles were set to 0.3, 0.5, and 0.7, respectively. Each simulated dataset consisted of two features to be visualized in a two-dimensional space because data visualization makes it easier to understand the distribution of case difficulty.

Different simulated datasets were generated for the three different metrics of measuring case difficulty (described in the Case Difficulty Metrics section below). Case difficulty model complexity (CDmc) used 2000 simulated cases for binary classification and 3000 simulated cases for 3-class classification (1000 per class). Case difficulty double model (CDdm) used 8000 and 12,000 simulated cases for binary and 3-class classification, respectively (4000 per class). Case difficulty predictive uncertainty (CDpu) used the same simulated datasets as CDmc.

The real-world datasets were chosen from three different domains: health, telecommunications, and marketing. The health data employed in this study were the UCI Wisconsin Breast Cancer Original data (UCI breast cancer data) from the UCI machine learning repository^12,13^. This is a binary classification dataset that includes 458 benign and 241 malignant breast cancer cases. The data consisted of nine features including clump thickness, uniformity of cell size, uniformity of cell shape, marginal adhesion, single epithelial cell size, bare nuclei, bland chromatin, normal nucleoli, and mitoses. Each feature was assigned an integer value between 1 and 10. The data included 16 missing values in the bare nuclei column denoted by ‘?’. These missing values were imputed with the mean values of the bare nuclei column. The standard scaler, a method that centers the data around 0 with a standard deviation of 1, was used to scale the data^11^.

The telecommunications data utilized in this study were the Telco Customer Churn data (Telco data) from the Kaggle dataset^14^. This is a binary classification dataset that involves customer information with the label column indicating whether a customer left within the last month. The dataset comprised 7043 instances, consisting of 1869 churned customers and 5174 non-churned customers. The data contained nineteen features including gender, senior citizen, partner, dependents, tenure, phone service, multiple lines, internet service, online security, online backup, device protection, tech support, streaming TV, streaming movies, contract, paperless billing, payment method, monthly charges, and total charges. There were 11 missing values in the total charge column, which occurred when the tenure value was 0, signifying that the customer used 0 months of service from the telco company. Thus, the missing values in the total charge column were replaced with 0. The data underwent preprocessing using a standard scaler for the continuous features (tenure, total charges, and monthly charges), while one-hot encoding was applied to the categorical features.

The marketing dataset was the Customer Segmentation data (Customer data) from Kaggle^15^. This is a multiclass classification dataset having 8068 instances categorized into four different customer groups: 1972 instances in group A, 1858 instances in group B, 1970 instances in group C, and 2268 instances in group D. The data had nine features including gender, ever married, age, graduated, profession, work experience, spending score, family size, and an anonymized variable. There were varying numbers of missing values in the following features: ever married (140 missing values), graduated (78 missing values), profession (124 missing values), work experience (829 missing values), family size (335 missing values), and the anonymized variable (76 missing values). These missing values were imputed using the most frequent values. The data were preprocessed using a standard scaler for continuous features (age, work experience, and family size), while one-hot encoding was applied to the categorical features.

### Case difficulty metrics

We developed and investigated three case difficulty metrics which are described in detail below.

### CDmc (case difficulty model complexity)

**Figure 1:**
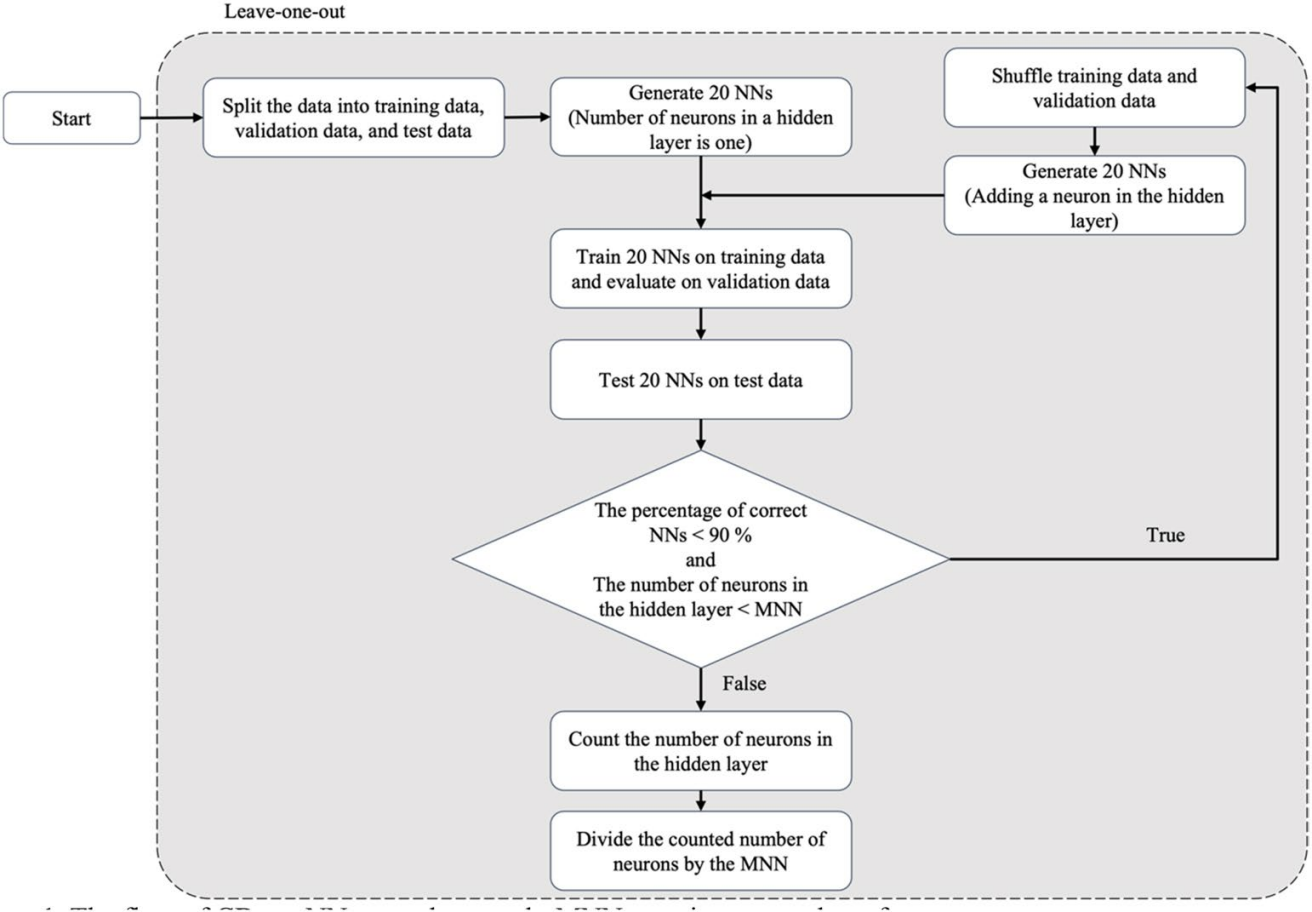
The flow of CDmc. *NN*: Neural network; *MNN*: Maximum number of neurons.

CDmc assumes that difficult cases require complex models to be predicted correctly. We counted the number of neurons an NN with one hidden layer would need to make a correct prediction. The flow of CDmc is shown in Fig. 1.

In Fig. 1, the NNs model began with the simplest structure: one neuron in one hidden layer. The NNs model used the ReLU activation function and the Adam optimizer. The batch size was set to 32 with 100 epochs and without dropout. The process started with one sample being left out as test data (i.e., leave-one-out). The rest of the data was split into 70% training and 30% validation. Twenty (arbitrarily chosen and can be changed depending on the problem or dataset) NNs were trained using training data with random initialization and using validation data for early stopping. If fewer than 90% of the 20 NNs could correctly predict the left-out test case, the model complexity was increased by adding a neuron. The process repeated until at least 90% of the 20 NNs could correctly predict the test case or reach a maximum number of neurons (MNN). This MNN was required since there was a chance that the model could not make a correct prediction regardless of the number of neurons. Therefore, the MNN was set as 1% of the sample size and used as a normalization factor in the metric. When the loop stopped, the number of neurons at that point was divided by the MNN. The calculated value was the case difficulty measure which ranged between 0 and 1, with an easy case being close to 0 and a difficult case being close to 1.

### CDdm (case difficulty double model)

**Figure 2:**
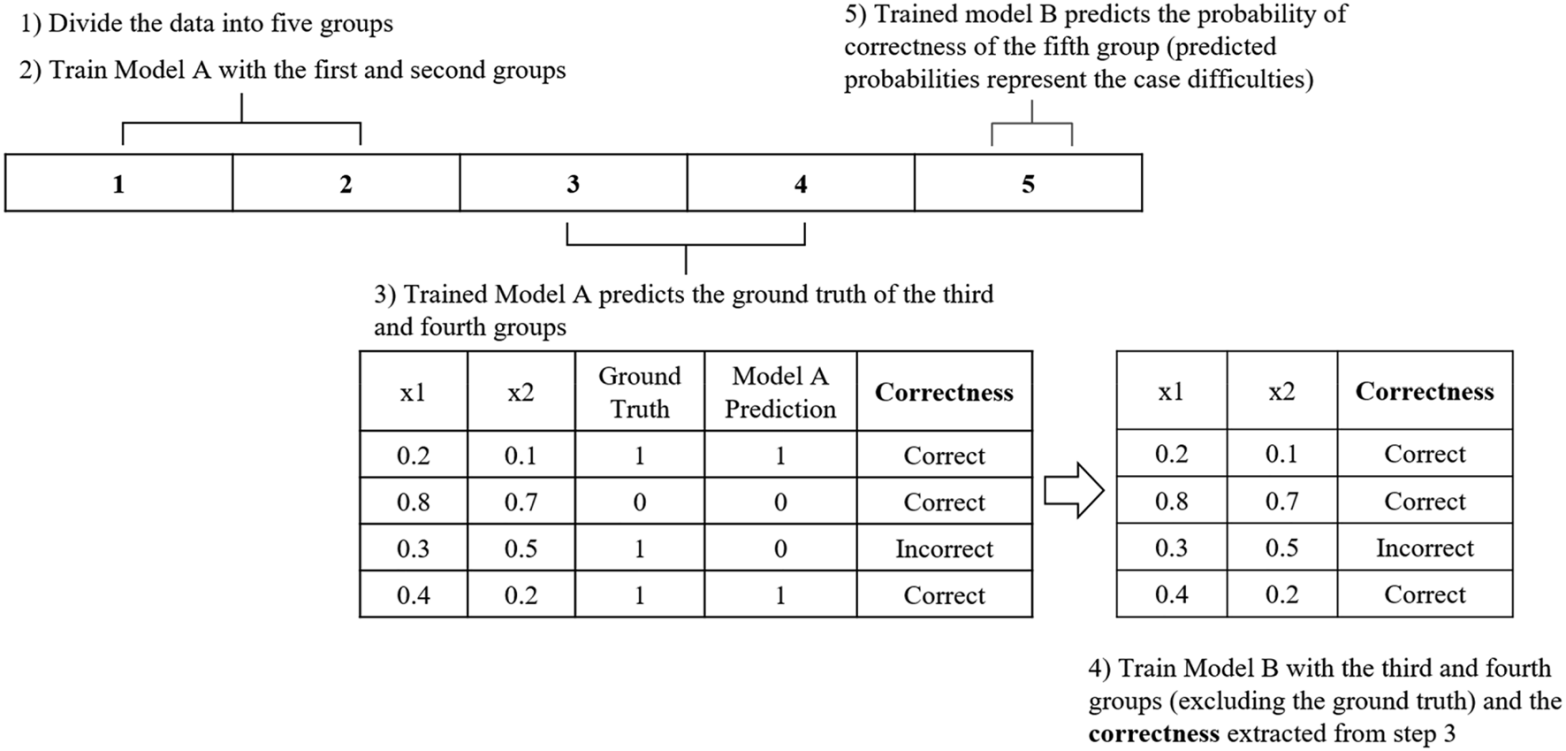
The flow of CDdm. Models A and B are neural networks. × 1 and × 2: example data in the third and fourth groups.

CDdm assumes that the prediction correctness of a model for a given case can be predicted by another model. The flow of CDdm is shown in Fig. 2.

First, data were divided into five sets of equal size. Next, Model A was trained on the first and second sets. Next, the trained model A predicted the cases in the third and fourth sets. Then, Model B was trained with a target variable (Correctness in Fig. 2) defined as whether Model A made correct or incorrect predictions. This allowed model B to predict the probability of correctness from the given data. Lastly, trained Model B made probability predictions of correctness for the fifth set. These predicted probabilities from Model B were the case difficulty of the individual cases in the fifth group. This process was repeated five times to obtain the case difficulties of all sets. Since predicted probabilities have a range between 0 and 1, the case difficulty values also range between 0 and 1, with values closer to 1 indicating more difficult cases.

Models A and B were NNs and Hyperopt was used for hyperparameter tuning. Hyperopt is a Python library for hyperparameter optimization which uses a form of Bayesian optimization to find the best hyperparameter settings^16^. Hyperopt searched in the following hyperparameter space: learning rate: 0.01, 0.03, 0.1; batch size: 32, 64, 128; number of hidden layers: 1, 2, 3; number of neurons in the hidden layer: 5, 10, 15, 20; and activation function: ReLU, Tanh. Both Models A and B were trained using the Adam optimizer and early stopping with 500 epochs and 50 patience. In Hyperopt, Models A and B were subject to 5 and 10 iterations, respectively, to find the best hyperparameter settings for the simulated data and UCI breast cancer data. For the Telco data and Customer data, since they were larger and more complex than the simulated data, Models A and B were subject to 200 and 200 iterations, respectively, to find the best hyperparameter settings.

### CDpu (case difficulty predictive uncertainty)

**Figure 3:**
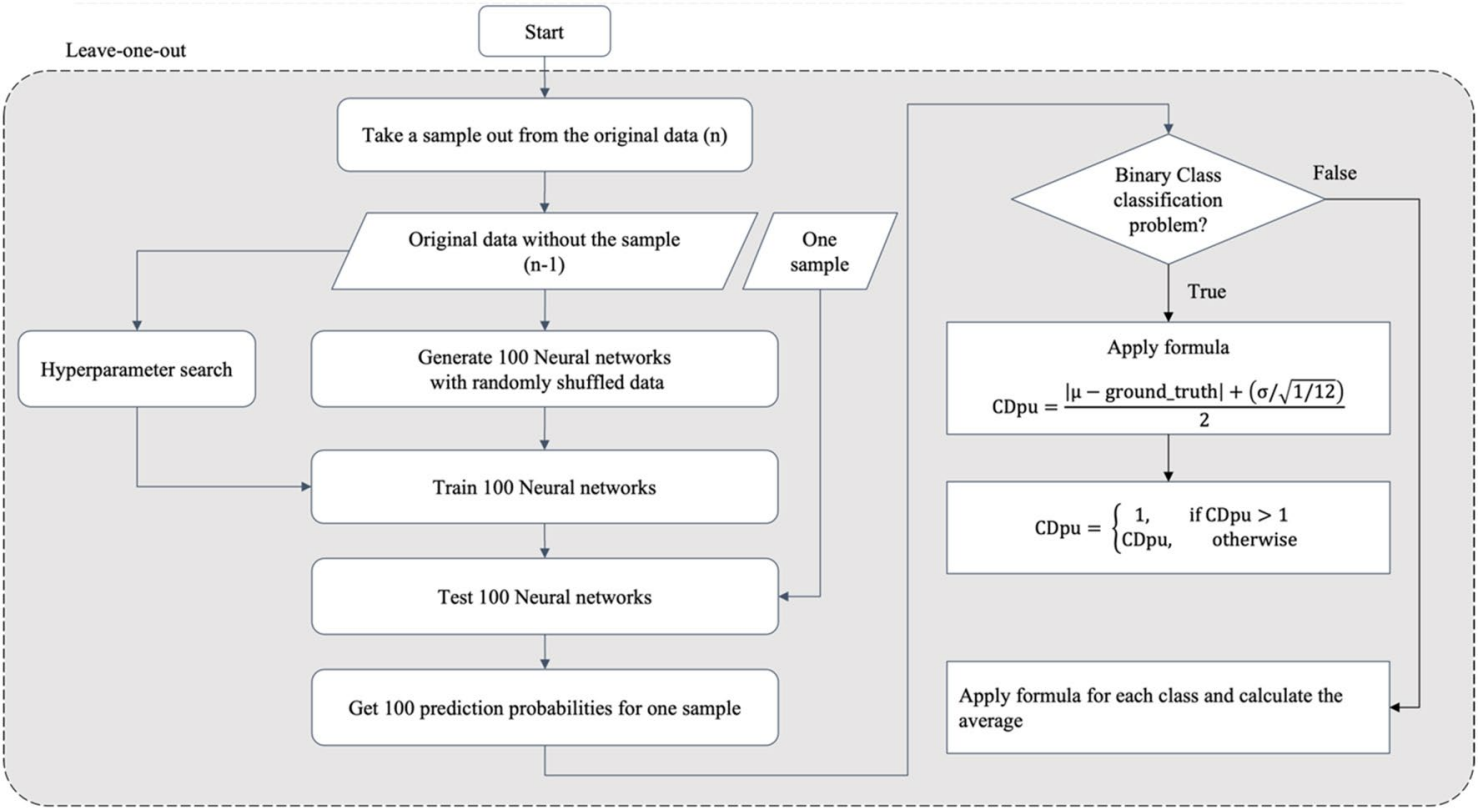
The flow of CDpu. $\mu$: mean value of prediction probabilities; ground truth: the target label of the sample that is excluded in the first step; $\sigma$: standard deviation of prediction probabilities.

CDpu defines case difficulty as the variability of predictions made by the model. The main assumption is that easy cases would lead to narrow prediction probability distributions (i.e., less uncertainty) near the correct label. Conversely, difficulty cases would lead to wide prediction probability distributions centered far from the correct label. We used the mean and standard deviation of the prediction probability distribution to calculate the case difficulty. The flow of CDpu is shown in Fig. 3.

The process began by leaving out one sample as test data (i.e., leave-one-out). The remaining data was divided into 70% for training and 30% for validation, aiming to tune the hyperparameters of a NN. 100 NNs were trained using a randomly shuffled original dataset, excluding the test data. Next, each of the 100 trained NNs generated a prediction probability for the test data. The formula in Fig. 3 was applied to the 100 prediction probabilities and the resulting value represented the case difficulty, with an easy case being closer to 0 and a difficult case being closer to 1.

The formula in Fig. 3 was developed based on the assumption that the worst prediction probability distribution is the uniform distribution. The uniform distribution represents the most conservative uncertainty estimation, and the standard deviation of the uniform distribution can be used as the normalization factor^17^. Since the prediction probability value ranged between 0 and 1, the standard deviation of the uniform distribution is approximately the square root of one over twelve. When the 100 predicted probabilities formed the distribution, the standard deviation of the prediction probabilities was calculated and divided by the normalization factor. The normalized value was the distribution factor. The distance from ground truth (the location factor) was calculated as the distance between the mean of the prediction probability distribution and ground truth (0 or 1). The average of this distance and the distribution factor became the case difficulty of the test data. For multi-class classification, the distribution and location factors were calculated for each class using the predicted probabilities according to each class. The average of the case difficulty values across all classes became the case difficulty of the test data.

There are rare cases when the case difficulty exceeds 1. This happens when the prediction probabilities exhibit a bimodal distribution pattern for 0 and 1, with two peaks in the distribution. Since the bimodal distribution results in a higher standard deviation than a uniform distribution, it can cause the distribution factor to go over 1. In such instances, the case difficulty is capped at 1.

To reduce computational time, Hyperopt ran under ray tune, which performs distributed hyperparameter tuning^18^. In Hyperopt, NNs were subject to 100 iterations. To find the best hyperparameter settings, Hyperopt searched in the following hyperparameter space: learning rate: 0.01, 0.03, 0.1; batch size: 32, 64, 128; number of hidden layers: 1, 2, 3; number of neurons in the hidden layer: 5, 10, 15, 20; and activation function: ReLU, Tanh. NN were trained using the Adam optimizer and early stopping with 100 epochs and 30 patience. For the UCI breast cancer data, early stopping was set to 10 patience because of its small sample size.

### Evaluation methods and statistical analysis

CDmc, CDdm, and CDpu were assessed using two methods. First, we visually inspected the case difficulty calculated from the existing metrics and proposed metrics. The existing metrics were calculated using the Pyhard Python package^18^. Second, we computed the Pearson and Spearman correlations between the case difficulty measures from our metrics and the existing metrics in the literature. For example, for a simulated dataset comprising 3000 cases, we obtained 3000 case difficulty values from each of the existing and the proposed metrics. Then, we calculated the Pearson and Spearman correlations between these sets of values. Pearson correlation is a parametric measure of correlation assessing the linear relationship between two continuous variables, whereas Spearman is a non-parametric measure of correlation evaluating the monotonic relationship between two continuous or ordinal variables^20^. Since we are comparing our metrics with multiple existing metrics that were developed based on various methods to calculate case difficulty, it is uncertain whether the relationship between the case difficulty from our metrics and the existing metrics adheres to parametric (linear) or non-parametric (non-linear or monotonic) patterns. Therefore, we have employed both Pearson and Spearman correlations to investigate the relationships between the metrics. This approach allows us to account for potential linear and non-linear associations and provides a more comprehensive analysis of the relationships.

## Results

### Simulated datasets

**Figure 4:**
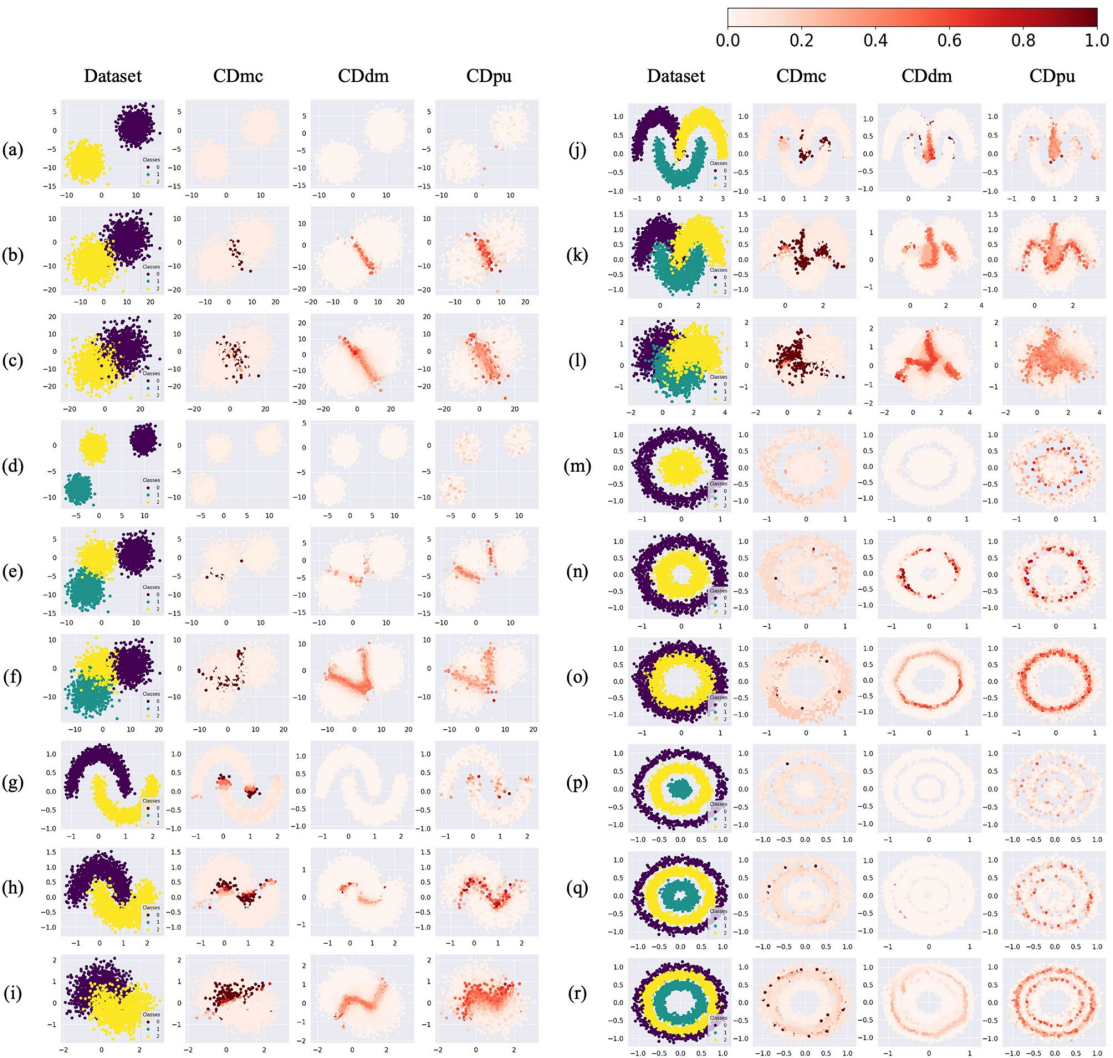
Case difficulty for the simulated datasets. The letters for the rows refer to various simulated datasets, while the columns represent the proposed metrics used to calculate the case difficulty (*CDmc*: Case difficulty model complexity, *CDdm*: Case difficulty double models, *CDpu*: Case difficulty predictive uncertainty). CDdm results were calculated using four times more samples than CDmc and CDpu because more training data were required to train two models. Case difficulty ranges from 0 to 1, with an easy case being colored light red and a hard case being colored dark red.

The case difficulty values for simulated datasets from CDmc, CDdm, and CDpu are plotted in Fig. 4. Figure 4 shows that difficult cases are mostly located in overlapping and borderline areas.

We selected one metric from each similarly developed group among the 15 existing metrics. The chosen metrics were kDN, DCP, TD_U, CL, CB, N1, LSC, and F1. The case difficulty values from selected simulated datasets are shown in Fig. 5 (See the Supplementary Figures S1-S18 for the results from the 15 existing metrics applied to the 18 simulated datasets).

**Figure 5:**
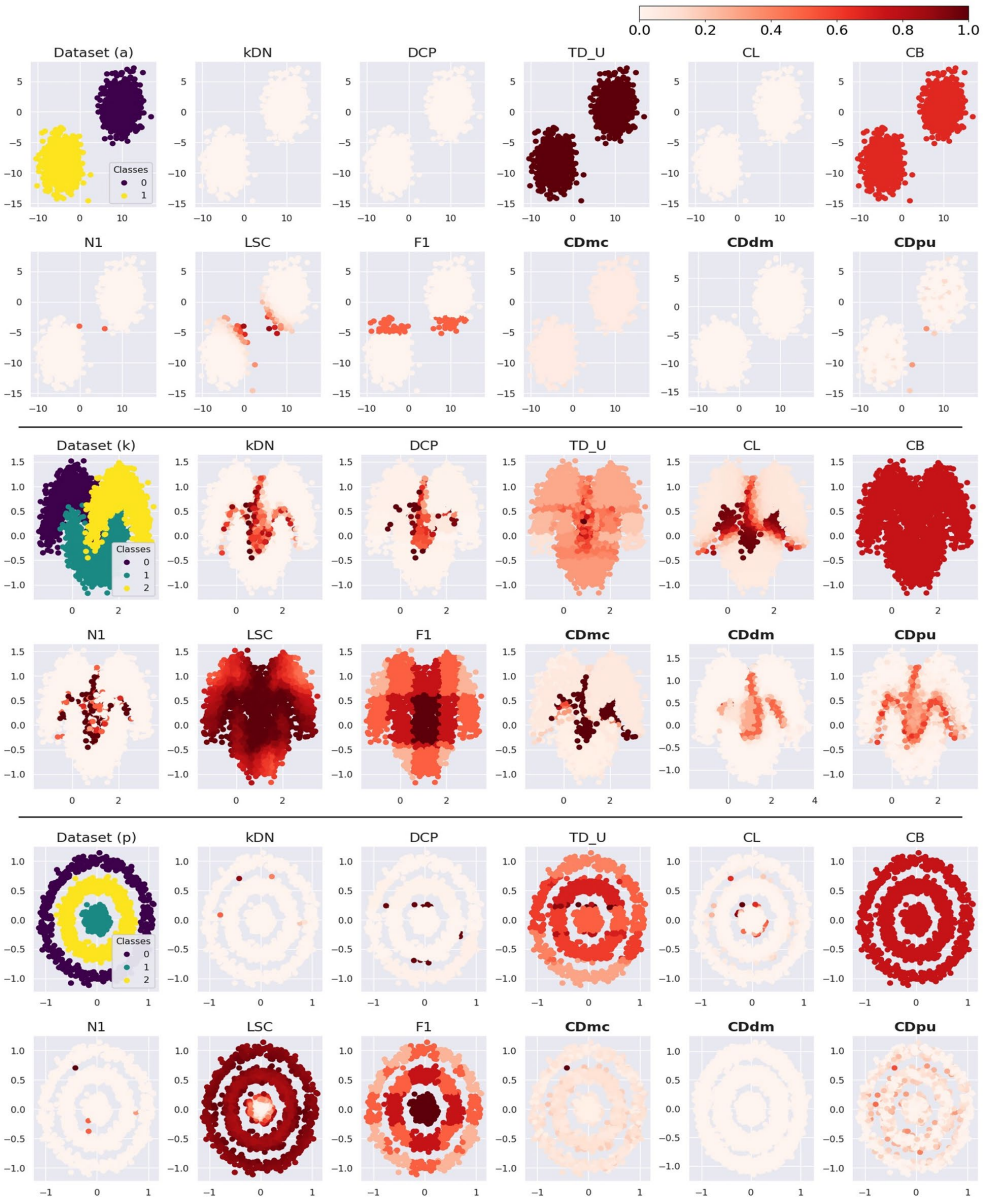
Case difficulty from the existing metrics (*kDN*: k-Disagreeing Neighbors, DCP: Disjunct Class Percentage, TD_U: unpruned decision trees, CL: Class likelihood, *CB*: Class balance, N1: Fraction of nearby instances of different classes, *LSC*: Local set cardinality, *F1*: Fraction of features in overlapping areas) and the proposed metrics (*CDmc*: Case difficulty model complexity, *CDdm*: Case difficulty double models, *CDpu*: Case difficulty predictive uncertainty). The dataset (a), (k), and (p) represent binary classification data with isotropic Gaussian blobs shape, multi-class classification data with interleaving crescent moons shape, and multi-class classification data with a large circle containing a smaller circle shape. CDdm results were calculated using four times more samples than CDmc and CDpu because more training data were required to train two models. Case difficulty ranges from 0 to 1, with an easy case being colored light red and a hard case being colored dark red.

Dataset (a) in Fig. 5 shows the results of the metrics applied to linearly separable data without overlapping areas. Most metrics indicate that all the cases have low case difficulty, while TD_U and CB show high case difficulty for every case. N1, LSC, F1, and CDpu show more diversely distributed case difficulty.

Dataset (k) in Fig. 5 shows the results of metrics applied to non-linearly separable data with overlapping areas. kDN, DCP, and N1 exhibit high case difficulty on the borderlines of the overlapped area. Similarly, CL, CDmc, CDdm, and CDpu display more scattered difficult cases along the borderline of the overlapped area. TD_U result expresses an attempt to classify the classes using linear lines, resulting in a distribution of case difficulty that follows linear lines. LSC and F1 present the highest case difficulty at the center of the data due to the overlap of the three classes.

Dataset (p) in Fig. 5 shows the results of metrics applied to non-linearly separable data without overlapping areas. kDN, DCP, N1, and CL exhibit a few challenging cases around the borderlines. TD_U and F1, however, do not effectively find the difficulty in the borderline area. CDdm demonstrates low case difficulty for every case. On the other hand, CDmc and CDpu reveal high case difficulty along the borderline of the simulated datasets of concentric circles, while LSC shows high difficulty cases for all cases except the center area. CB could not differentiate difficulty levels in datasets (a), (k), and (p) since these datasets were balanced between target classes.

**Figure 6:**
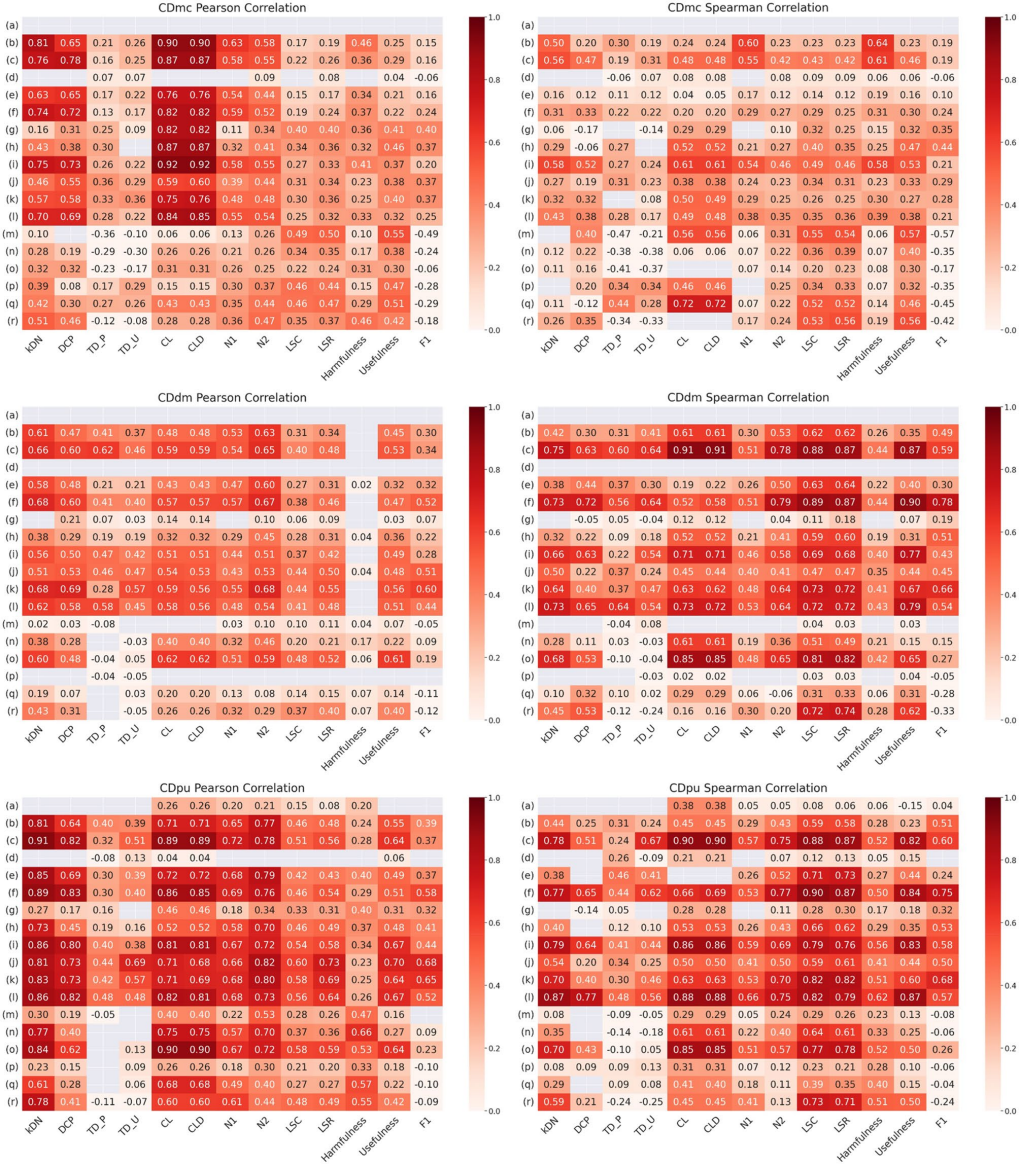
Correlations between the case difficulty values from the proposed metrics (*CDmc*: Case difficulty model complexity, *CDdm*: Case difficulty double models, *CDpu*: Case difficulty predictive uncertainty) and the existing metrics (*kDN*: k-Disagreeing neighbors, *DCP*: Disjunct class percentage, TD_P: Pruned decision trees, TD_U: Unpruned decision trees, *CL*: Class likelihood, *CLD*: Class likelihood difference, *N1*: Fraction of nearby instances of different classes, N2: Ratio of intra/extra class nearest neighbor distance, *LSC*: Local set cardinality, *LSR*: Local set radius, harmfulness, usefulness, *F1*: Fraction of features in overlapping areas) for the simulated datasets. Only the correlation values with a *p*-value below 0.05 are displayed. Each row corresponds to each simulated dataset described in Fig. 4, and the columns are described by the acronyms of the existing metrics described in Table 1. The MV and CB are not shown since the simulated datasets had balanced classes and these metrics could not be calculated. The colors represent the strength of correlation. The correlation color ranges between 0 and 1, with a weak correlation colored light red and a strong positive correlation colored dark red.

The Pearson and Spearman correlations between our metrics and the existing metrics are shown as heatmaps in Fig. 6.

Figure 6 shows the case difficulty from CDmc has a higher correlation when evaluated using the Pearson correlation than the Spearman correlation. The results show measures from metrics kDN, DCP, CL, CLD, and N1 show the highest correlations with CDmc. Moreover, lower correlations occurred when the data were simulated datasets of concentric circles. For instance, case difficulties ranging from simulated data (m) to (r) exhibited lower correlations than the correlations in the other simulated datasets.

Case difficulty from CDdm is more correlated with the existing metrics when evaluated by the Spearman correlation method. Most existing metrics showed a high positive correlation with the case difficulty from CDdm. In particular, higher correlations occurred when the data had more overlapping areas. For example, case difficulties from simulated data (c) resulted in higher correlations than those from simulated data (b), and case difficulties from simulated data (f) showed higher correlations than those from simulated data (e).

Case difficulty from CDpu has a higher correlation when evaluated using the Pearson correlation. Similar to CDmc, the case difficulty from CDpu demonstrates the higher correlation with existing metrics such as kDN, DCP, CL, CLD, and N1. However, CDpu also shows a high correlation with N2 and higher correlations with the existing metrics for the simulated datasets of concentric circles (m) to (r).

### Real-world data

**Figure 7:**
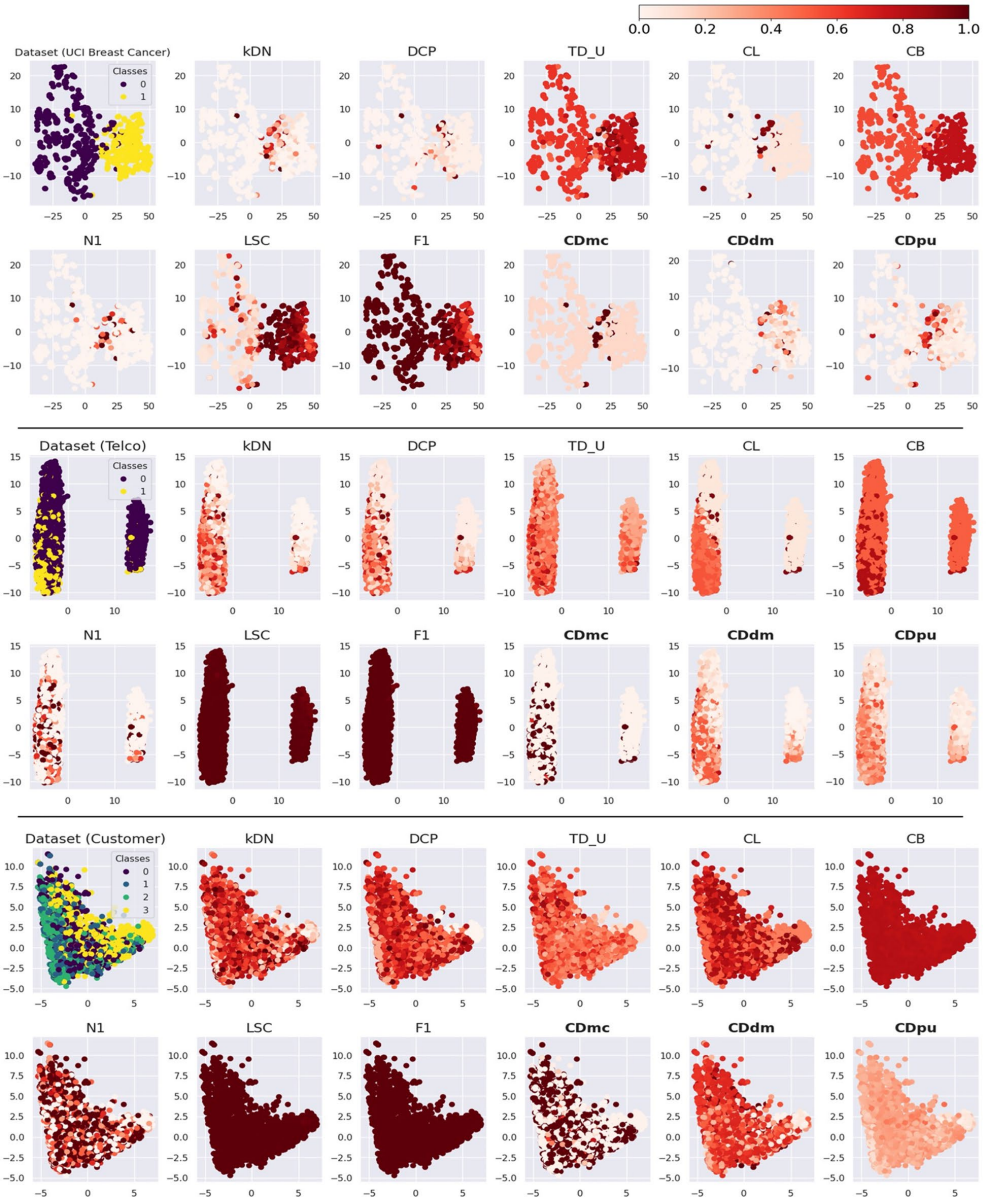
Case difficulty from the existing metrics (*kDN*: k-disagreeing neighbors, *DCP*: Disjunct class percentage, *TD_U*: Unpruned decision trees, *CL*: Class likelihood, *CB*: Class balance, *N1*: Fraction of nearby instances of different classes, LSC: Local Set Cardinality, F1: Fraction of features in overlapping areas) and proposed metrics (*CDmc*: Case difficulty model complexity, *CDdm*: Case difficulty double models, *CDpu*: Case difficulty predictive uncertainty). The t-SNE was used to visualize the UCI breast cancer data, and the FAMD was used to visualize the Telco data and Customer data. The number of principal components was set to 2 to plot the results in a two-dimensional space. The case difficulty scale ranges from 0 to 1, with the lightest color indicating a low case difficulty, and the darkest color indicating a high case difficulty.

UCI breast cancer data was unable to be plotted as a two-dimensional image due to its nine features. Therefore, we applied t-distributed Stochastic Neighbour Embedding (t-SNE) to reduce the dimensionality and enable visualization^19^. The UCI breast cancer data were standardized before applying t-SNE. Similarly, Telco data and Customer data could not be plotted as two-dimensional images due to their nineteen and nine features. Since these datasets contain both categorical and continuous features, the Factor Analysis of Mixed Data (FAMD) was used to reduce the dimensionality and allow visualization^20^. The case difficulty results of UCI breast cancer data, Telco data, and Customer data are plotted in Fig. 7 (See the Supplementary Figures S19-S21 for the results from the 15 existing metrics applied to the real-world data).

UCI breast cancer data in Fig. 7 shows that kDN and N1 are similar to CDmc, demonstrating high case difficulty in the overlap area and outliers on the left side. CDpu, DCP, and CL assigned high case difficulty for the cases in the overlap area and several outliers. TD_U, CB, LSC, F1, and CDdm were more influenced by the class imbalance and assigned high case difficulties to the minor class.

Telco data in Fig. 7 shows that kDN, DCP, CDdm, and CDpu share similar difficulty distributions. TD_U displayed a widely distributed case difficulty in the upper left area, whereas CL exhibited concentrated high case difficulty in the lower left area. CB assigned high case difficulty to the minor class due to the class imbalance. N1 and CDmc depicted high case difficulty at the borderline of the overlapped area, while LSC and F1 struggled to differentiate the difficulty of individual cases.

Customer data in Fig. 7 shows that the Customer data exhibit substantial overlap between the target classes. The central overlapping area was effectively recognized by kDN, DCP, TD_U, CL, N1, CDmc, CDdm, and CDpu. In contrast, CB, LSC, and F1 were inadequate to be used with the Customer data. Furthermore, the clusters of low case difficulty were observed in two areas. Dataset (Customer) in Fig. 7 shows that class 2 cases are linearly clustered on the left side, where DCP, CL, CDmc, and CDpu successfully captured these patterns and indicated lower difficulty in these areas. On the middle right side, round-shaped class 3 cases are clustered, which were well identified by kDN, DCP, TD_U, CL, N1, CDmc, CDdm, and CDpu.

**Figure 8:**
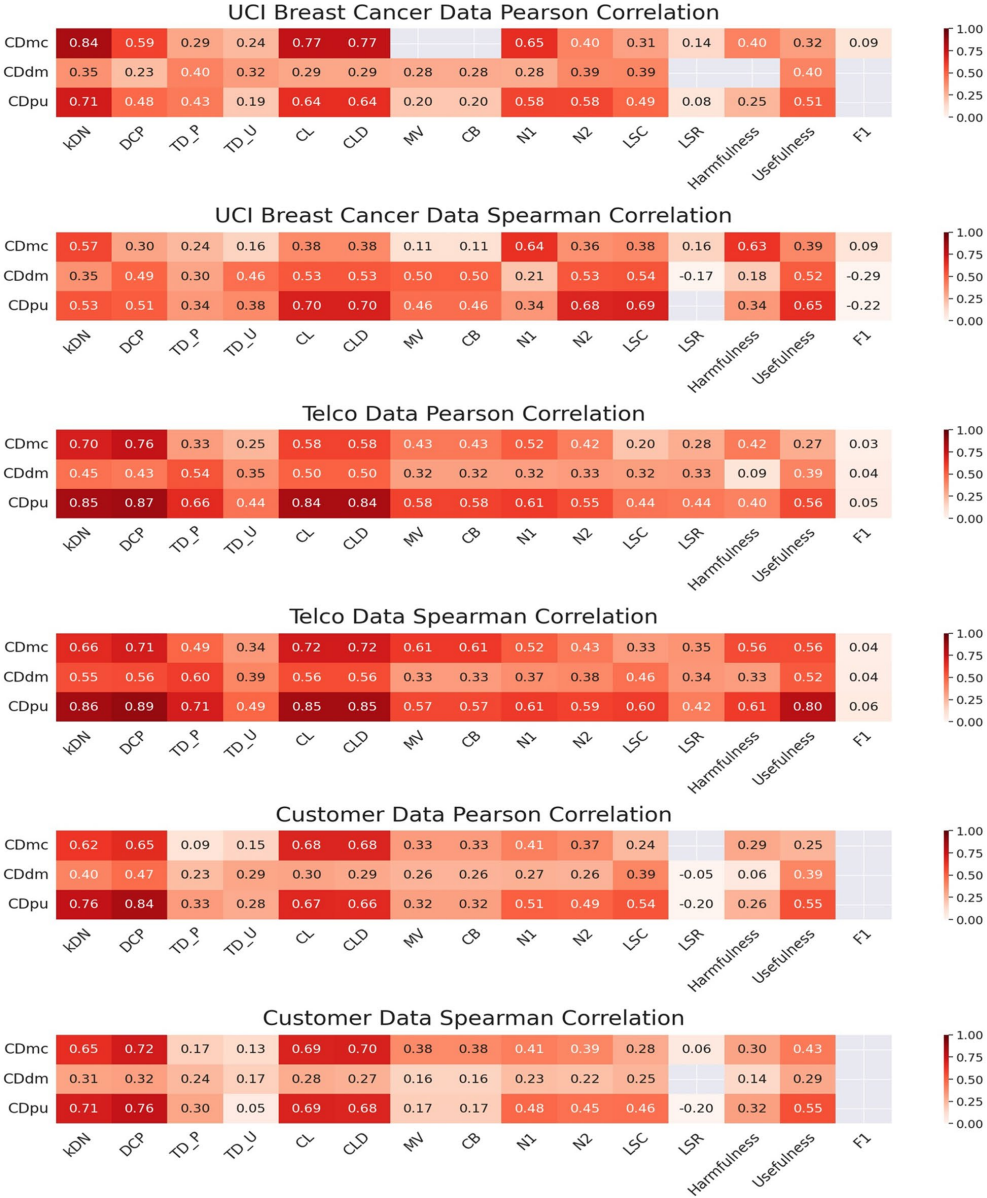
Correlation between case difficulty from the existing metrics (*kDN*: k-disagreeing neighbors, *DCP*: Disjunct class percentage, *TD_P*: Pruned decision trees, *TD_U*: Unpruned decision trees, *CL*: Class likelihood, *CLD*: Class likelihood difference, *MV*: Minority value, *CB*: Class balance, *N1*: Fraction of nearby instances of different classes, *N2*: Ratio of intra/extra class nearest neighbor distance, *LSC*: Local set cardinality, *LSR*: Local set radius, harmfulness, usefulness, *F1*: Fraction of features in overlapping areas) and the proposed metrics (*CDmc*: Case difficulty model complexity, *CDdm*: Case difficulty double models, *CDpu*: Case difficulty predictive uncertainty) for the real-world datasets. Only the correlation values with a *p*-value below 0.05 are displayed. The rows correspond to the proposed metrics used to calculate the case difficulty, and the columns are the existing metrics described in Table 1. The colors represent the strength of the association. The darker red color represents a strong correlation, while the lighter red color means a weak correlation.

The computed correlations with the existing metrics are shown in Fig. 8.

Figure 8 shows case difficulty from CDmc, CDdm, and CDpu mostly have a positive correlation with the existing metrics.

UCI breast cancer data in Fig. 8 shows the proposed metrics were closely related to the existing metrics in the order of CDmc, CDpu, and CDdm. When the correlation is evaluated with the Pearson correlation method, case difficulty from CDmc showed a stronger association with the existing metrics. When the correlation was evaluated with the Spearman correlation method, case difficulty from CDdm and CDpu showed a stronger association with the existing metrics.

Telco data in Fig. 8 shows the proposed metrics were closely linked to the existing metrics in the following order: CDpu, CDmc, and CDdm. When the correlations were evaluated with the Spearman correlation method, the case difficulty from CDmc, CDdm, and CDpu showed stronger associations with the existing metrics.

Customer data in Fig. 8 shows the proposed metrics were highly related to the existing metrics in the order of CDpu, CDmc, and CDdm. When the correlation is evaluated with the Pearson correlation method, case difficulty from CDdm and CDpu showed a stronger association with the existing metrics. Conversely, when the correlation is evaluated with the Spearman correlation method, case difficulty from CDmc showed a stronger association with the existing metrics.

## Discussion

This study aimed to develop new case difficulty metrics showing good performance for a wide range of different datasets. The existing metrics in the literature require specific dataset preconditions, limiting their applicability to certain datasets. However, our case difficulty metrics perform well across diverse datasets and can provide a unique perspective for understanding difficulty. Furthermore, we evaluated our metrics using real-world data from various domains and successfully verified their performance across datasets from different domains. Comparisons between the existing metrics and CDmc, CDdm, and CDpu are summarized in Table 2. The existing metrics were executed on a system with Intel(R) Xeon(R) CPUs @ 2.20 GHz and 13 GB RAM. The computational times for the UCI breast cancer data in Table 2 were measured when the CDmc used 20 CPUs, CDdm used 1 CPU, and CDpu used 30 CPUs with 10 GB RAM (Intel(R) Xeon(R) Gold 6342 CPUs @ 2.80 GHz).

**Table 2:** Comparison between the existing metrics and CDmc, CDdm, and CDpu.

Method	Computational time (UCI brest cancer data)	Class balanced data	No class overlapped data	Mixed features data	Dependent features	Non-linearly separable data	Required data size	Need ground truth
Neighborhood- based method (kDN)	< 1 min	Good	Medium	Poor	Good	Good	Medium	Yes
Decision tree-based method (DCP, TD_P, TD_U)	< 1 min	Good	Medium	Good	Good	Poor	Medium	Yes/No
Naïve bayes-based method (CL, CLD)	< 1 min	Good	Good	Good	Poor	Good	Medium	Yes
Class skewness-based method (MV, CB)	< 1 min	Poor	Good	Good	Good	Good	Small	Yes
Distance-based method (N1, N2, LSC, LSR, Harmfulness, Usefulness)	< 1 min	Good	Medium	Medium	Good	Good	Medium	Yes
Feature-based method (F1)	< 1 min	Good	Medium	Poor	Good	Good	Medium	Yes
Case Difficulty Model Complexity (CDmc)	~ 6 h	Good	Good	Good	Good	Good	Big	Yes
Case Difficulty Double Model (CDdm)	~ 10 min	Good	Good	Good	Good	Good	Big	No
Case Difficulty Predictive Uncertainty (CDpu)	~ 5 h	Good	Medium	Good	Good	Good	Big	Yes

Table 2 demonstrates that the current metrics have drawbacks when encountering specific data conditions. This may be an important point to consider in some applications. For example, the neighborhood-based method showed vulnerability when dealing with data containing continuous and categorical features. With Telco and Customer data, the neighborhood-based method failed to identify several easy cases. Since this method heavily relies on the nearest data points, the presence of both numeric and categorical features could lead to degraded performance of the neighborhood-based method.

Decision tree-based methods are vulnerable to non-linearly separable data. The results showed the metrics poorly performed for interleaving crescent moons shapes and data shapes with a large circle containing smaller circles. The metrics need deeper trees to understand non-linear patterns, and the lack of modification of the decision tree's parameters caused lower performance.

The Naïve Bayes classification-based methods have a premise that features need to be independent. The metric did not work well in identifying the case difficulty of Telco data since the data have dependent features such as monthly and total charges.

The class skewness-based methods are inappropriate for balanced data. When the simulated datasets are balanced, Class skew-based methods could not use class imbalance information to calculate the case difficulty.

The distance-based methods showed varying performance across different datasets since these methods encompass various distance calculation metrics. N1 and N2 yield good performance in most datasets, while LSC, LSR, Harmfulness, and Usefulness often show poor performance based on the dataset. Particularly, most methods did not function well with the Telco and Customer data, which have mixed continuous and categorical features.

The feature-based methods did not perform well for either simulated or real-world data. Since the feature-based methods utilize the relationship between the features of a case and the overlap area, they may exhibit limited performance in calculating case difficulty when the dataset has no overlap area or when the dataset is too complex the classes to be separated using the features.

The proposed metrics (CDmc, CDdm, and CDpu) consistently demonstrated strong performance across various datasets. CDmc and CDpu process data individually, which leads to longer computational times but yields more reliable results. However, CDdm takes less computational time by processing data in groups. Moreover, CDdm has one of the notable advantages in that it can calculate the case difficulty of a new data point without knowing its ground truth.

Taking a closer look at the correlation comparison result, CDmc and CDpu displayed a higher correlation when evaluated using the Pearson correlation method, while CDdm showed a higher correlation with the Spearman correlation method. This finding suggests that the case difficulty from CDmc and CDpu had a linear relationship with the measures from the previous studies and the case difficulty from CDdm had a monotonic relationship. In other words, case difficulty from CDmc and CDpu changed proportionally with the measures from the previous studies, while case difficulty from CDdm showed a consistent pattern of change.

The results showed that CDmc, CDdm, and CDpu have a strong positive correlation with the measures from CL and CLD. The reason is that CL and CLD both use similar concepts to how NNs compute case difficulty. CL and CLD use the likelihood of an instance belonging to its class to measure the case difficulty^4^. Similarly, trained NNs make a prediction based on the probability of each case belonging to each class^21^. Therefore, the measures from these metrics and case difficulty from CDmc, CDdm, and CDpu can show a high correlation.

Likewise, kDN and DCP showed similar correlation values with the proposed metrics as they use a similar concept to calculate the case difficulty. The kDN metric determines the case difficulty by estimating the percentage of instances that do not belong to the same class based on 10 nearest neighbours^4^. Similarly, the DCP metric evaluates the percentage of the same class in the leaf node induced from the decision tree^4^. As the leaf node generally contains the nearest cases, kDN and DCP consider similar values to calculate the difficulty.

The results showed that measures from TD_P and TD_U have a low correlation for the simulated datasets of concentric circles. As the decision tree is inefficient to solve complex problems such as XOR, parity or multiplex problems, these metrics could not calculate proper case difficulty for these datasets^22,25^.

CDmc, CDdm, and CDpu showed a high correlation when dealing with more complex datasets, while no correlation results were available for simple datasets. When the dataset is simple, the existing metrics and metrics assigned the same lowest case difficulty to all cases. With the constant case difficulty, it was impossible to calculate either the Pearson correlation or Spearman correlation, resulting in the empty cells in rows (a) and (d) of Fig. 6.

### Limitations

There are several limitations with our case difficulty metrics and points to consider when deciding which metric to use. First, high computational resources are needed to implement CDmc, CDdm, and CDpu. Training NNs requires high computational power^23^, and the proposed metrics require many iterations of NN training and evaluation. CDmc requires training 20 NNs until 90% of them make the correct prediction or the MNN is reached. CDdm performed hyperparameter tuning for two NNs several times. CDpu is associated with the highest computational cost to train and hyperparameter-tune100 NNs for each case. However, case difficulty only needs to be calculated once and can be used again in other studies. Therefore, high computational costs may be acceptable. Furthermore, we used various techniques such as multiprocessor, Hyperopt, and ray tune to reduce computational time as much as possible.

Second, the number of NNs and MNN in CDmc are arbitrary and should be adjusted based on the sample size and complexity of the data. In this study, we utilized 20 NNs and set 1% of the sample size for MNN. Despite increasing the number of NNs and MNN, we found that CDmc produced similar results for the simulated data. However, the optimal number of NNs and MNN may vary depending on the data. When the data is more complicated, increasing the number of NNs can yield more stable results and increasing MNN can test more neurons in the hidden layer which might be needed to make a correct prediction. Similarly, increasing the number of NNs in CDpu did not significantly affect the results for the simulated data. Thus, appropriate adjustments to the number of NNs in the CDpu calculation may also be necessary.

Third, the hyperparameter range needs to be modified depending on the data. As the proposed metrics use NNs, hyperparameter tuning is essential^24^, particularly in CDdm. As the two NNs work together in CDdm, finding hyperparameters for both models are important. The first model's performance affects the second model's ability to determine the case difficulty. Although finding the best hyperparameters for NNs requires additional effort, NNs' capability to detect complex patterns in datasets makes them an effective tool for determining case difficulty.

Fourth, our metrics require sufficient sample sizes. Insufficient data can hinder the training of NNs and result in low performance^23,25^. This is especially important for CDdm, as the data is divided into five groups, resulting in fewer training data for NNs. We discovered that using the same number of simulated data from CDmc for CDdm was inadequate. Therefore, we increased the sample size of the simulated data to investigate CDdm. Despite that CDdm requires more data than CDmc, CDdm is preferred when the research needs more differentiated case difficulties.

Fifth, as the case difficulty from CDmc is directly related to the number of neurons, the resolution of the case difficulty metric can be limited when MNN is small. For instance, if MNN is 10, the case difficulty metric has a step size of only 1/10 = 0.1 which may be too coarse. In contrast, the case difficulty metric from CDdm is a probability and has a float value ranging from 0 to 1.

Sixth, the effect of data preprocessing on case difficulty was not analyzed. Data preprocessing can ensure data quality and improve a model’s performance. However, altering cases during preprocessing could affect their inherent difficulty. Therefore, we applied minimal preprocessing to the real-world data, which included only imputation for missing values and scaling for different features as required for NNs. This allowed us to preserve the individual cases in their original forms.

Seventh, uncertainty quantifications, such as confidence intervals or metric value ranges, were not included for the proposed metrics. In this study, we recorded a single value for case difficulty for each case, as it was the only value needed for comparisons between the proposed and existing metrics. However, incorporating uncertainty quantification may be necessary in some instances to enhance the reliability of these metrics.

Lastly, if there are more than two features, it becomes hard to assess the case difficulty results through visualization. In this study, the UCI breast cancer data were visualized using t-SNE, and Telco and Customer data were visualized using FAMD. But it is uncertain whether the dimensional reduced results can accurately represent the original data. Therefore, an additional evaluation method needs to be used. One possible evaluation method is to compute the correlation between the log-loss values of various ML models and case difficulty. Previous research has shown that model misclassification is related to case difficulty^4,5^. Therefore, when the data is complex and cannot be visualized as an image, comparing the log-loss values of various ML models can help to evaluate case difficulty.

### Future work

In future work, the design of the proposed metrics in this paper can be further extended using ML models other than NNs. Additionally, other factors that may affect the calculation or credibility of case difficulty could be explored. This exploration might include aspects mentioned here as limitations, such as data preprocessing or the presentation of uncertainty estimates. Furthermore, our metrics can be expanded to assess case difficulty in regression problems and used to develop novel prediction performance evaluation metrics. For the performance evaluation metrics, it is possible to apply the case difficulty values derived from the proposed metrics as weights. This can allow us to observe the changes in performance based on the difficulty of individual cases.

## Conclusions

In this paper, we proposed three novel case difficulty metrics based on model complexity, a double model, and predictive uncertainty. These metrics performed well across several datasets and required less data preconditions than existing metrics. These metrics were particularly effective when there were no overlapped areas in the dataset or when the dataset contained both categorical and continuous features. Moreover, the high correlation with some existing metrics indicates that the proposed metrics can capture case difficulty in a similar manner to the existing metrics. Despite these advantages, the proposed metrics have some limitations. Besides high computational complexity, the metrics need appropriate modifications based on the datasets and require sufficient sample sizes. Future work could involve using ML models other than NNs to address these limitations, extending the research to measure case difficulty in different types of data and develop new, case difficulty-aware prediction performance metrics. We expect using our case difficulty metrics can provide a new perspective to ML researchers in many fields and provide more detailed case-by-case explanations to users.

### Supplementary Information

Supplementary Information.

## Data Availability

The datasets generated and analysed during the current study are available in the GitHub repository, https://github.com/data-intelligence-for-health-lab/Measuring_Case_Difficulty.
