# Supplementary material for: Measuring the prediction difficulty of individual cases in a dataset using machine learning
Source: Sci Rep. 2024 May 7;14:10474. doi: 10.1038/s41598-024-61284-z (PMC11076552; doi:10.1038/s41598-024-61284-z)
Supplement: Supplementary file 1 — Supplementary Information. [file 41598_2024_61284_MOESM1_ESM.docx]

Appendix

The Appendix includes case difficulty results from the 15 existing metrics (kDN: k-Disagreeing Neighbors, DCP: Disjunct Class Percentage, TD_P: Pruned Decision Tree, TD_U: Unpruned Decision Tress, CL: Class Likelihood, CLD: Class Likelihood Difference, MV: Minority Value, CB: Class Balance, N1: Fraction of nearby instances of different classes, N2: Ratio of Intra/Extra Class Nearest Neighbor Distance, LSC: Local Set Cardinality, LSR: Local Set Radius, Harmfulness, Usefulness, F1: Fraction of features in overlapping area) and the 3 proposed metrics (CDmc: Case difficulty model complexity, CDdm: Case difficulty double models, CDpu: Case difficulty predictive uncertainty) applied to the 18 simulated datasets and 3 real-world datasets.


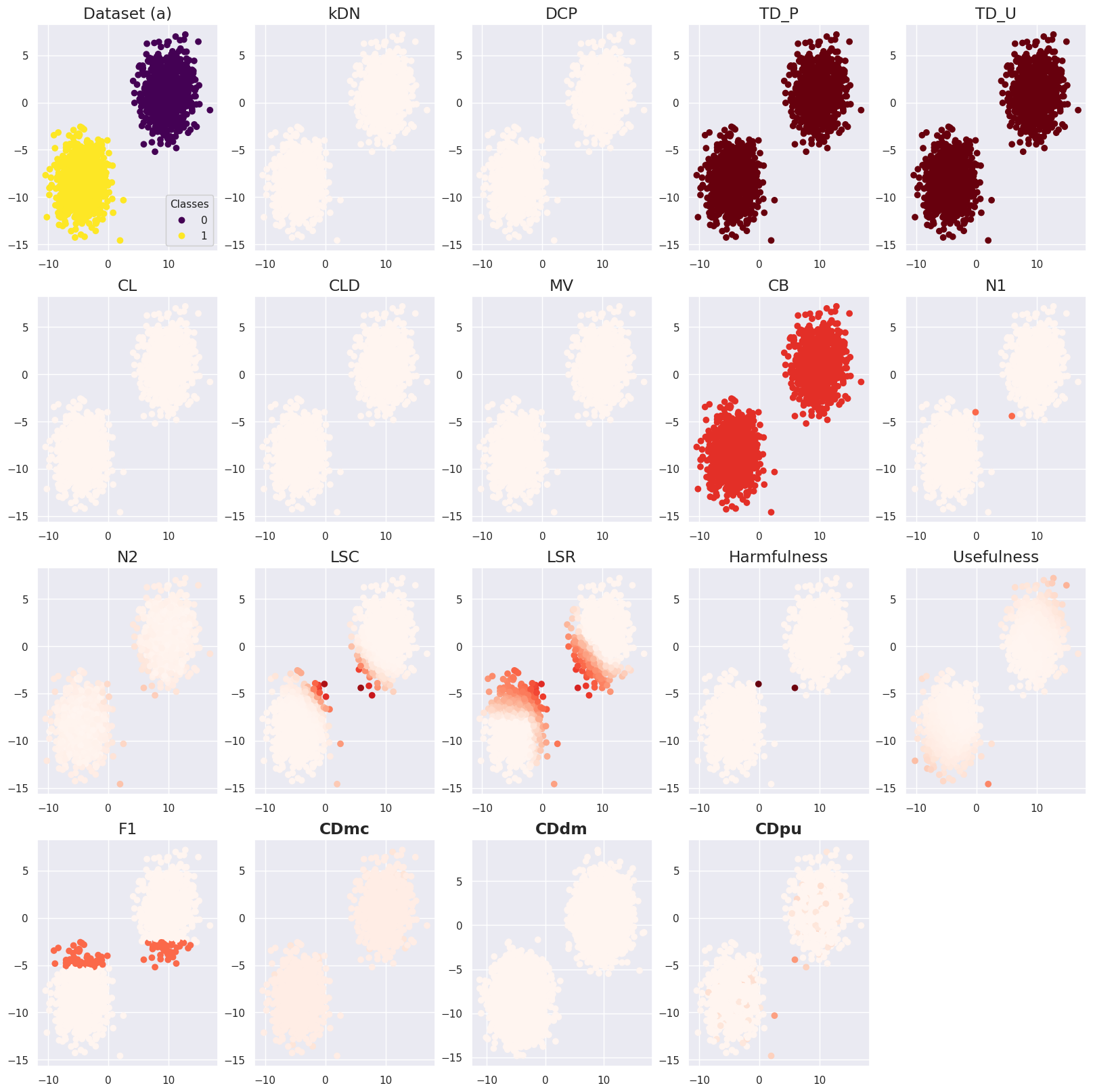


**Supplementary Figure S1.** Case difficulty of the dataset (a) from the existing metrics and the proposed metrics. CDdm results were calculated using four times more samples than CDmc and CDpu because more training data were required to train two models. Case difficulty ranges from 0 to 1, with an easy case being colored light red and a hard case being colored dark red.

**
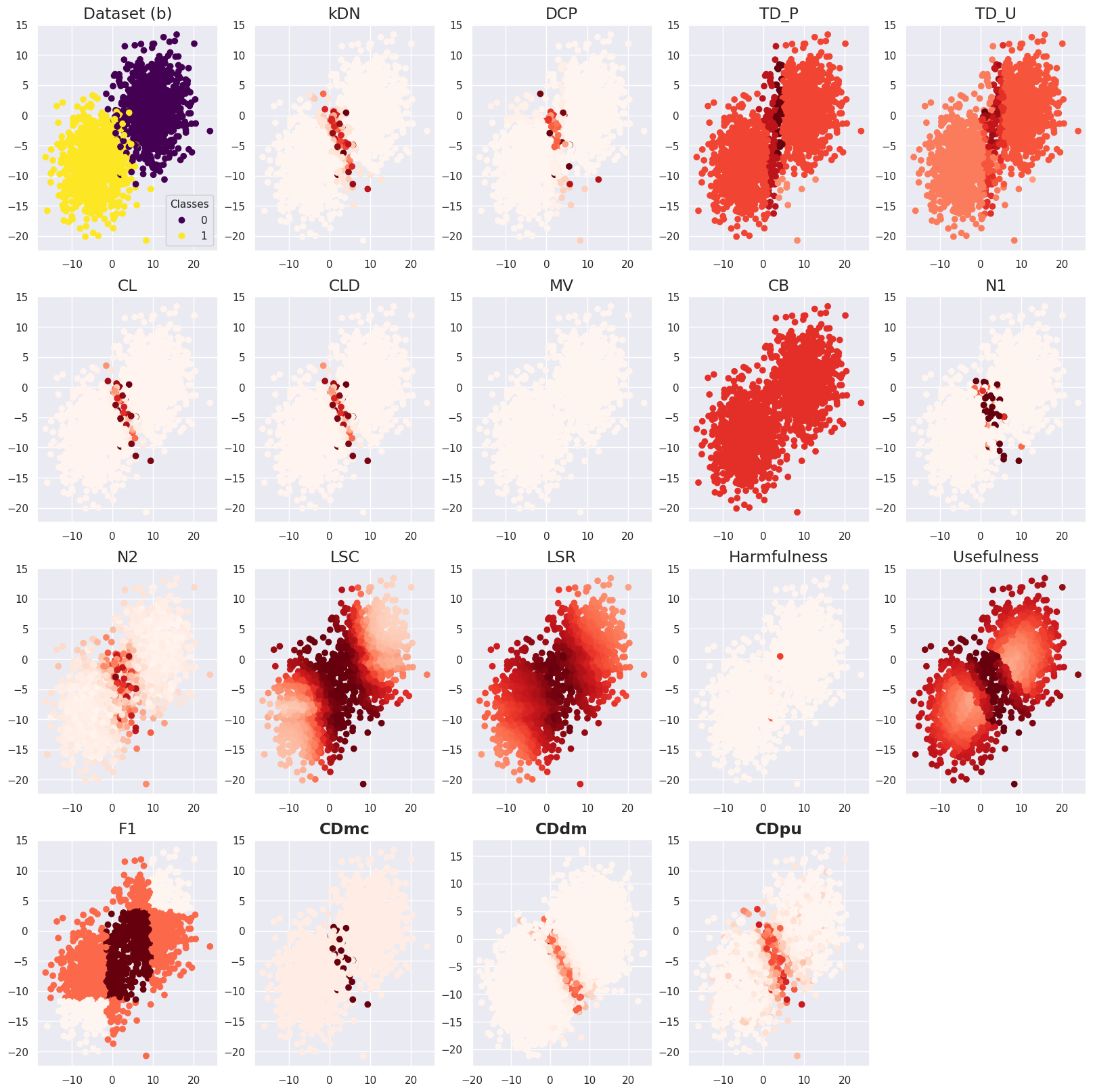
**

**Supplementary Figure S2.** Case difficulty of the dataset (b) from the existing metrics and the proposed metrics. CDdm results were calculated using four times more samples than CDmc and CDpu because more training data were required to train two models. Case difficulty ranges from 0 to 1, with an easy case being colored light red and a hard case being colored dark red.


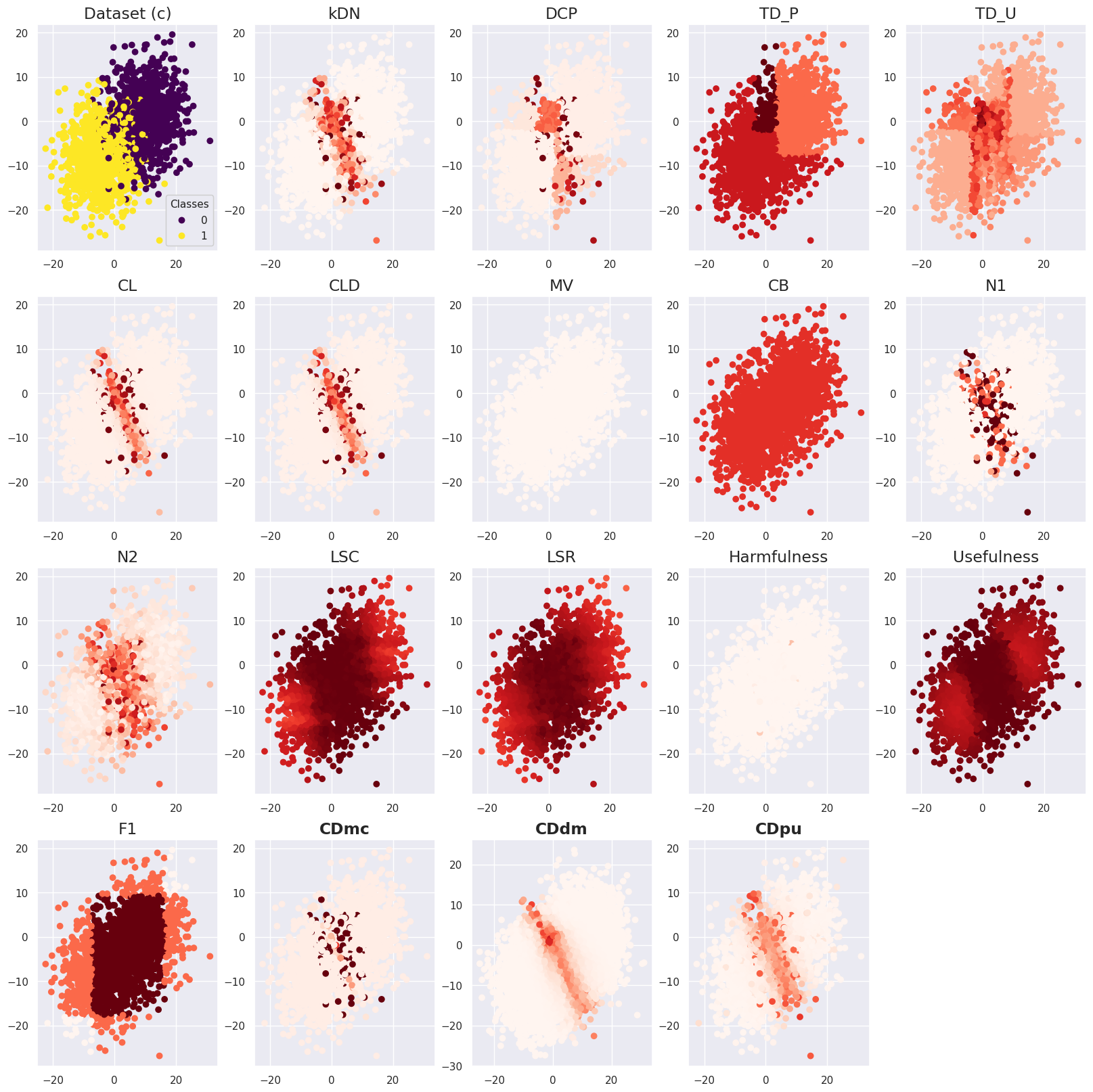


**Supplementary Figure S3.** Case difficulty of the dataset (c) from the existing metrics and the proposed metrics. CDdm results were calculated using four times more samples than CDmc and CDpu because more training data were required to train two models. Case difficulty ranges from 0 to 1, with an easy case being colored light red and a hard case being colored dark red.


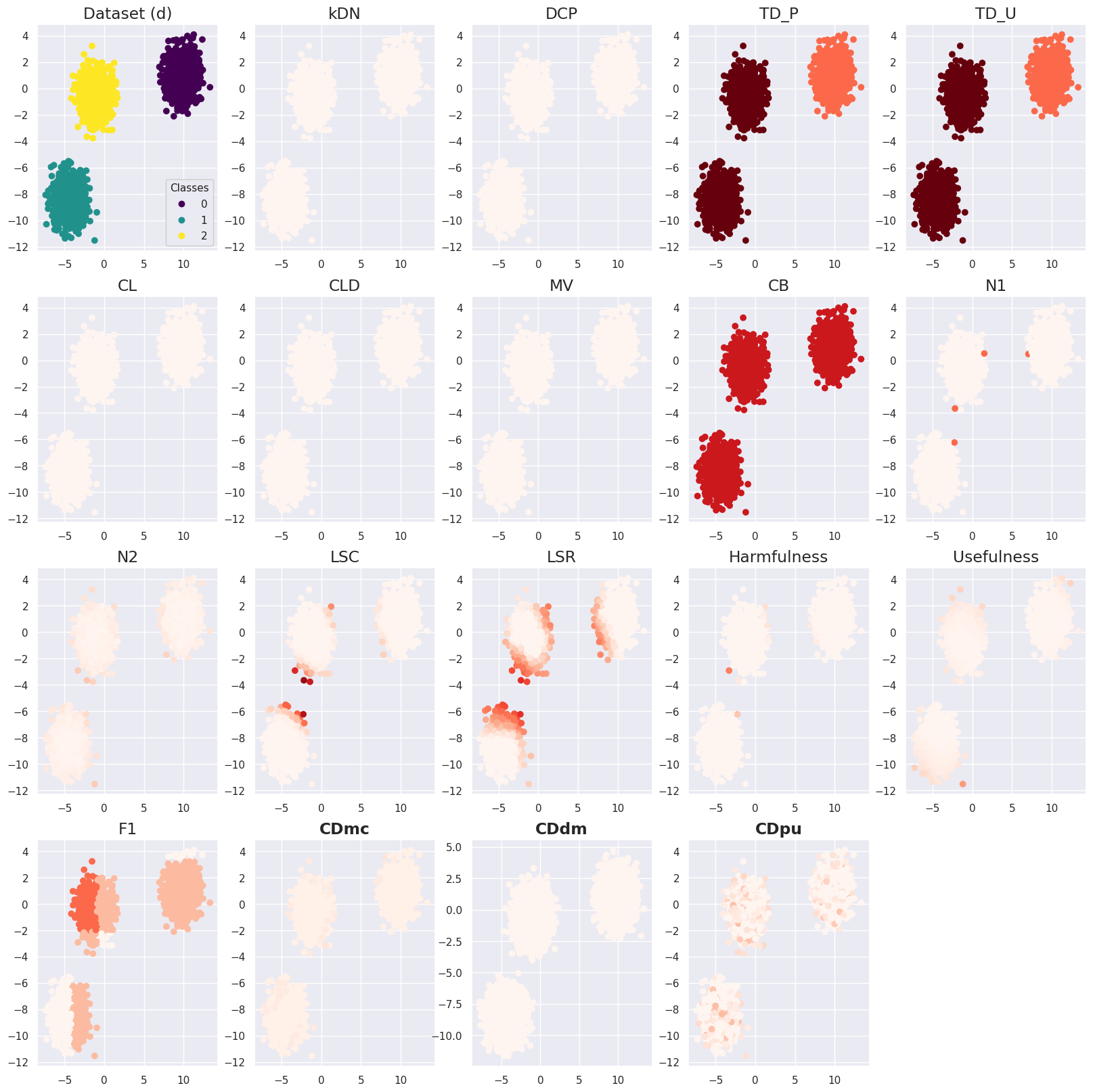


**Supplementary Figure S4.** Case difficulty of the dataset (d) from the existing metrics and the proposed metrics. CDdm results were calculated using four times more samples than CDmc and CDpu because more training data were required to train two models. Case difficulty ranges from 0 to 1, with an easy case being colored light red and a hard case being colored dark red.


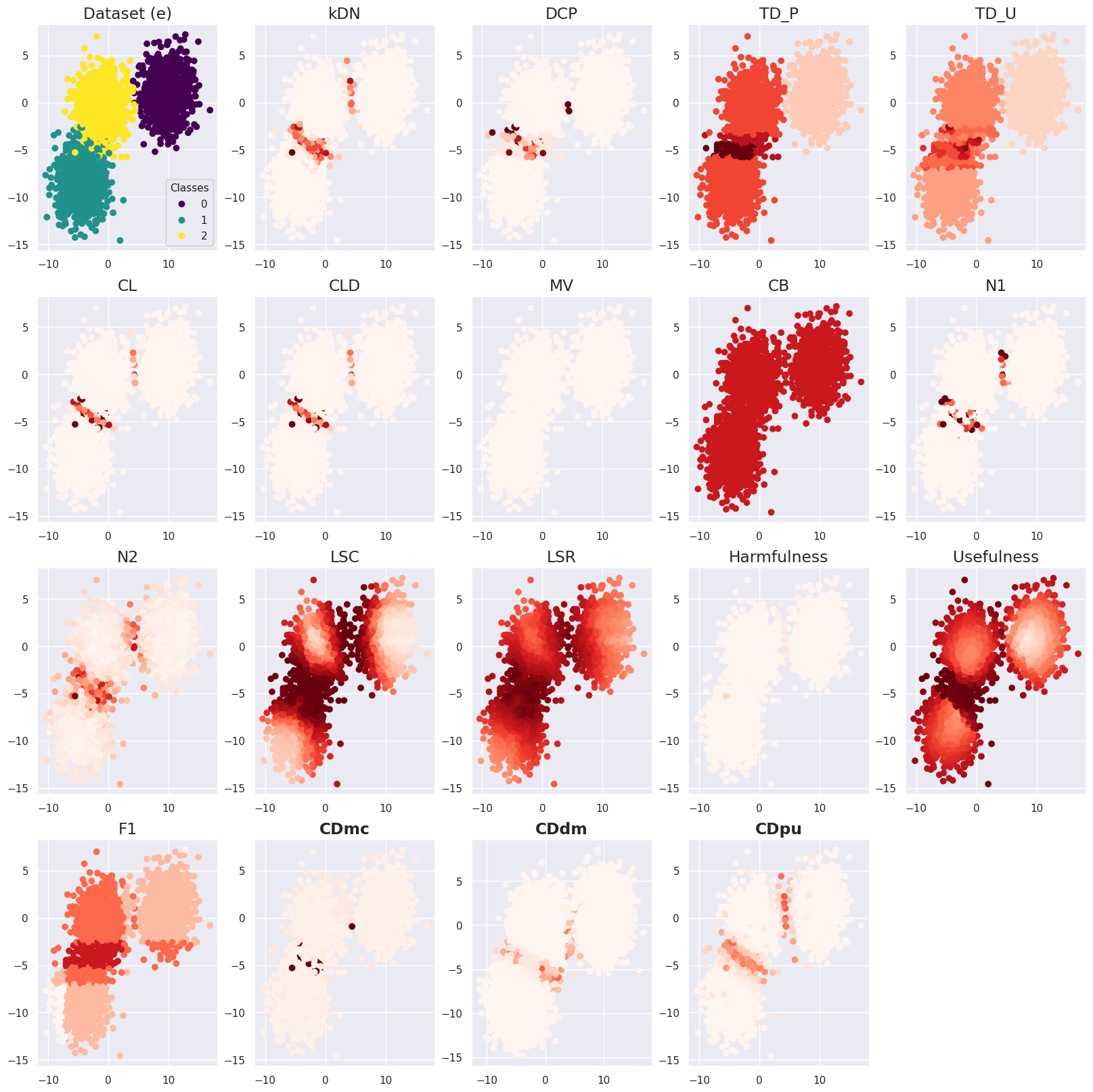


**Supplementary Figure S5.** Case difficulty of the dataset (e) from the existing metrics and the proposed metrics. CDdm results were calculated using four times more samples than CDmc and CDpu because more training data were required to train two models. Case difficulty ranges from 0 to 1, with an easy case being colored light red and a hard case being colored dark red.


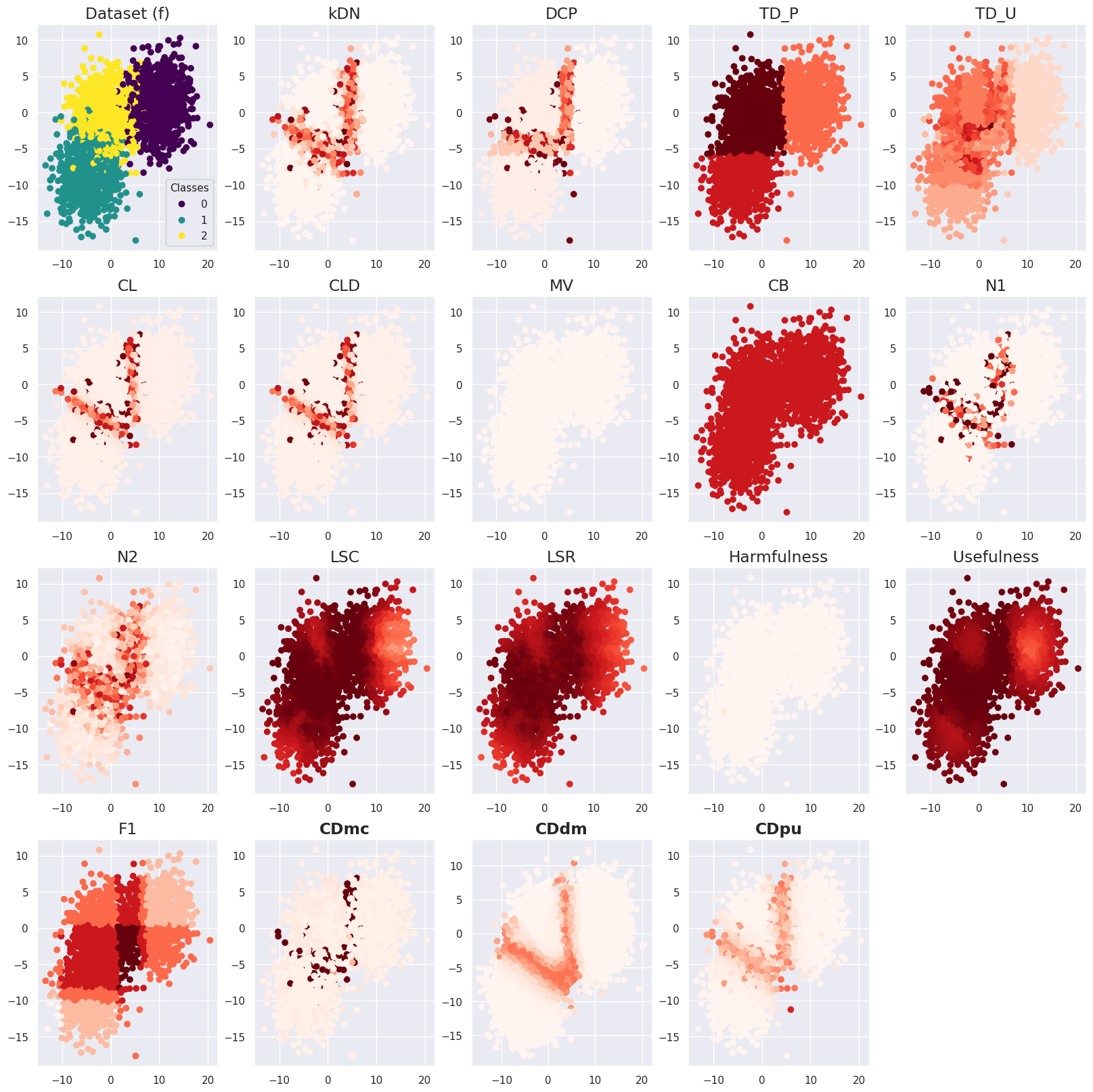


**Supplementary Figure S6.** Case difficulty of the dataset (f) from the existing metrics and the proposed metrics. CDdm results were calculated using four times more samples than CDmc and CDpu because more training data were required to train two models. Case difficulty ranges from 0 to 1, with an easy case being colored light red and a hard case being colored dark red.


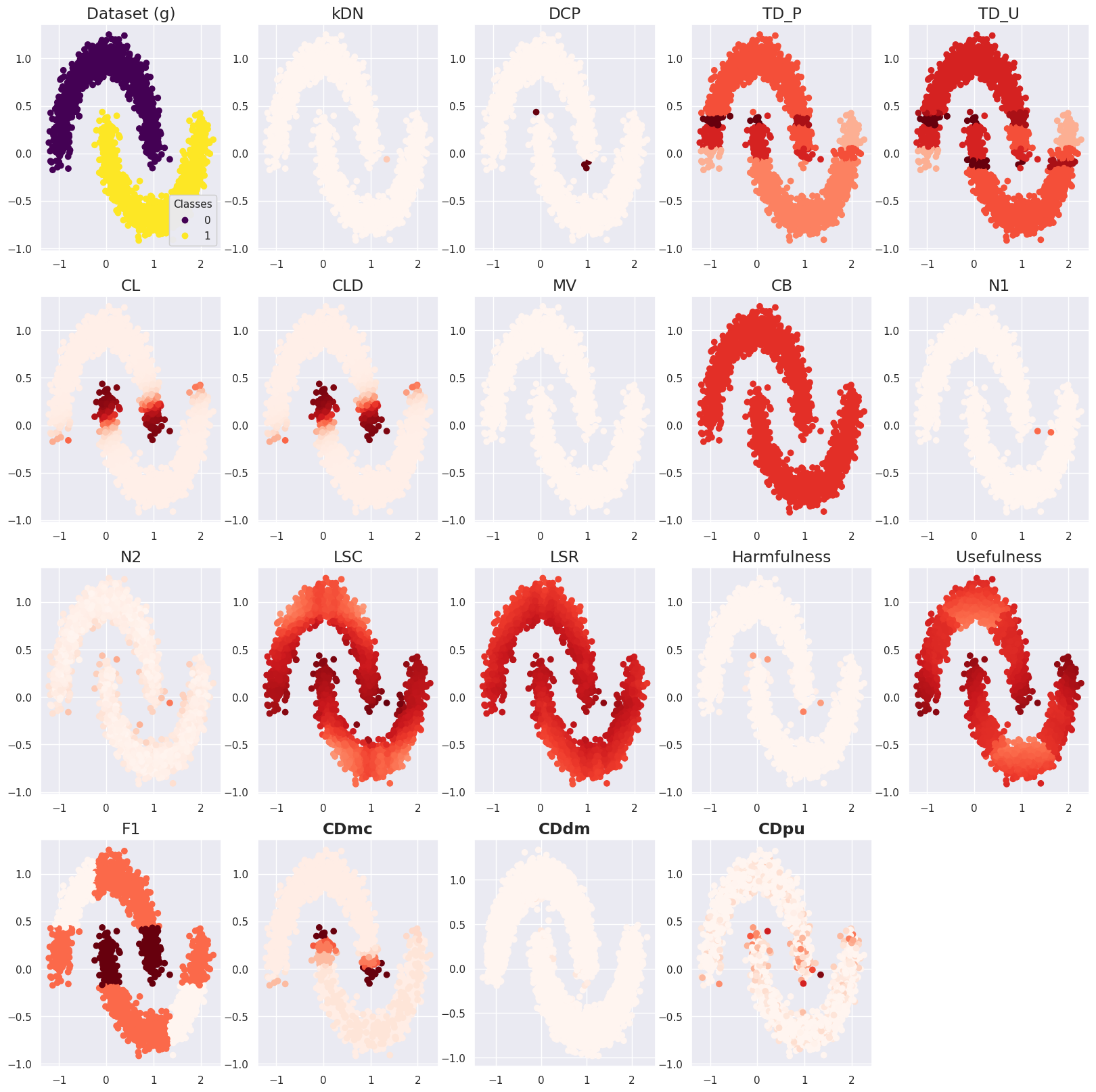


**Supplementary Figure S7.** Case difficulty of the dataset (g) from the existing metrics and the proposed metrics. CDdm results were calculated using four times more samples than CDmc and CDpu because more training data were required to train two models. Case difficulty ranges from 0 to 1, with an easy case being colored light red and a hard case being colored dark red.


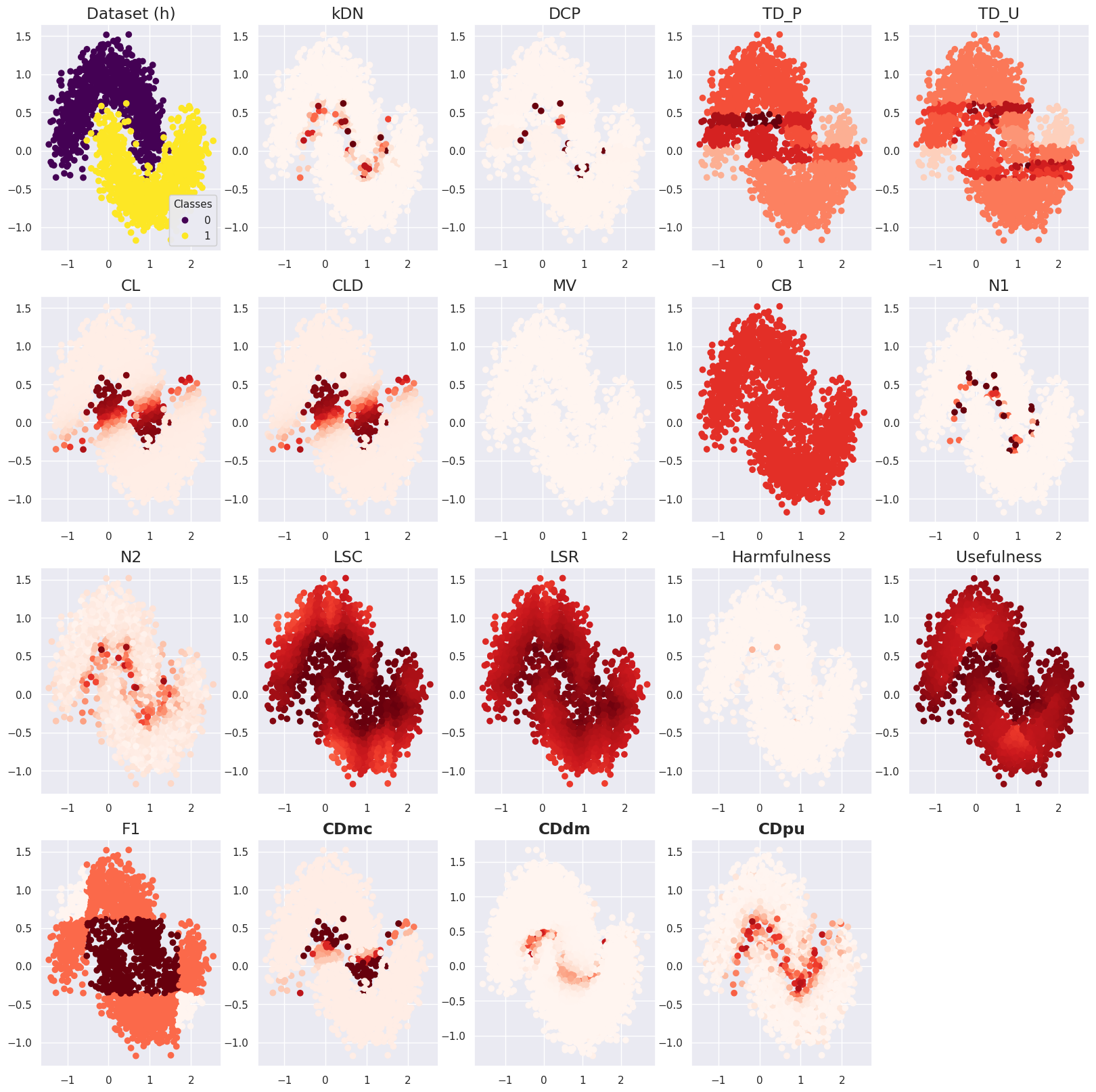


**Supplementary Figure S8.** Case difficulty of the dataset (h) from the existing metrics and the proposed metrics. CDdm results were calculated using four times more samples than CDmc and CDpu because more training data were required to train two models. Case difficulty ranges from 0 to 1, with an easy case being colored light red and a hard case being colored dark red.


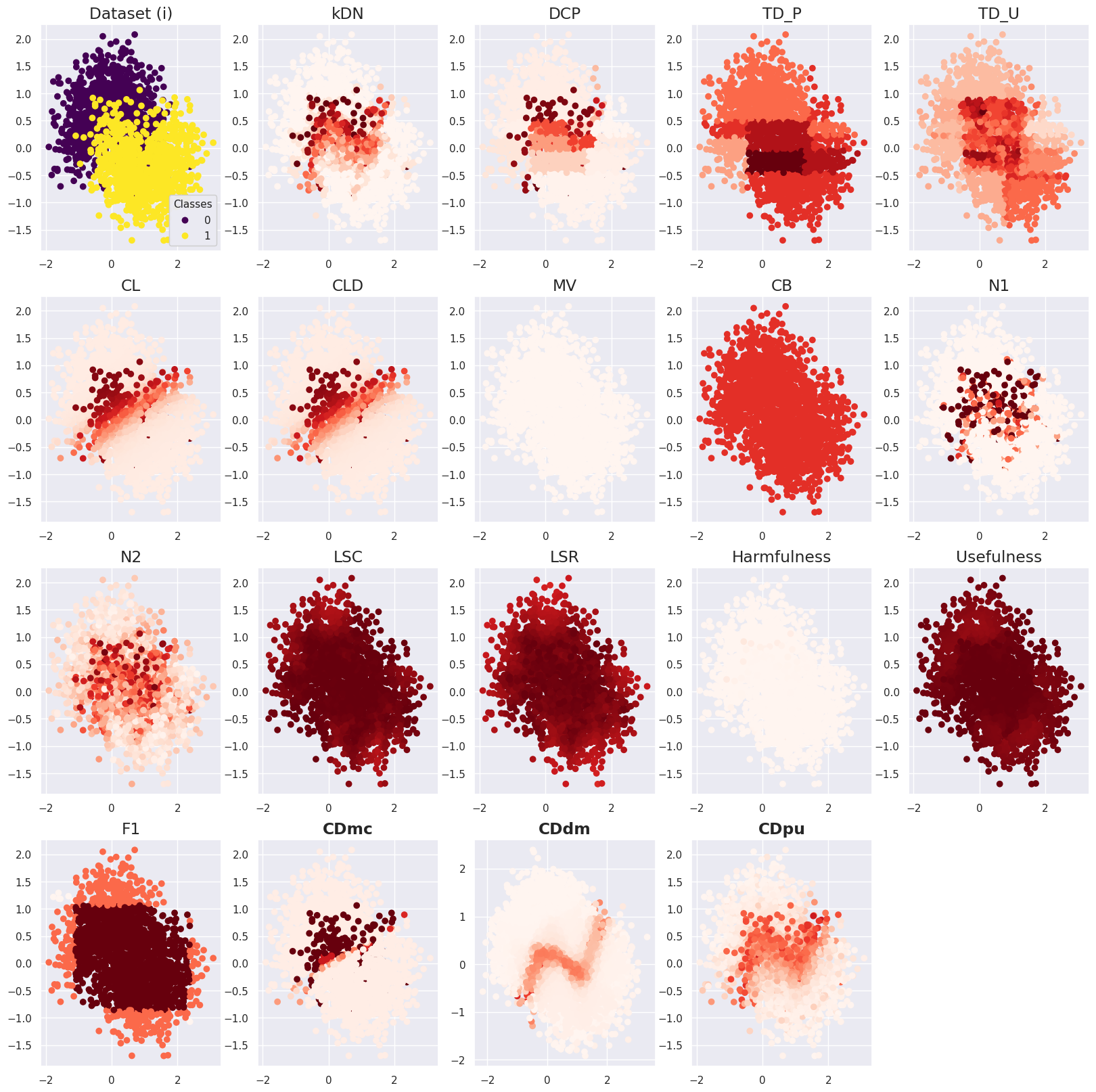


**Supplementary Figure S9.** Case difficulty of the dataset (i) from the existing metrics and the proposed metrics. CDdm results were calculated using four times more samples than CDmc and CDpu because more training data were required to train two models. Case difficulty ranges from 0 to 1, with an easy case being colored light red and a hard case being colored dark red.


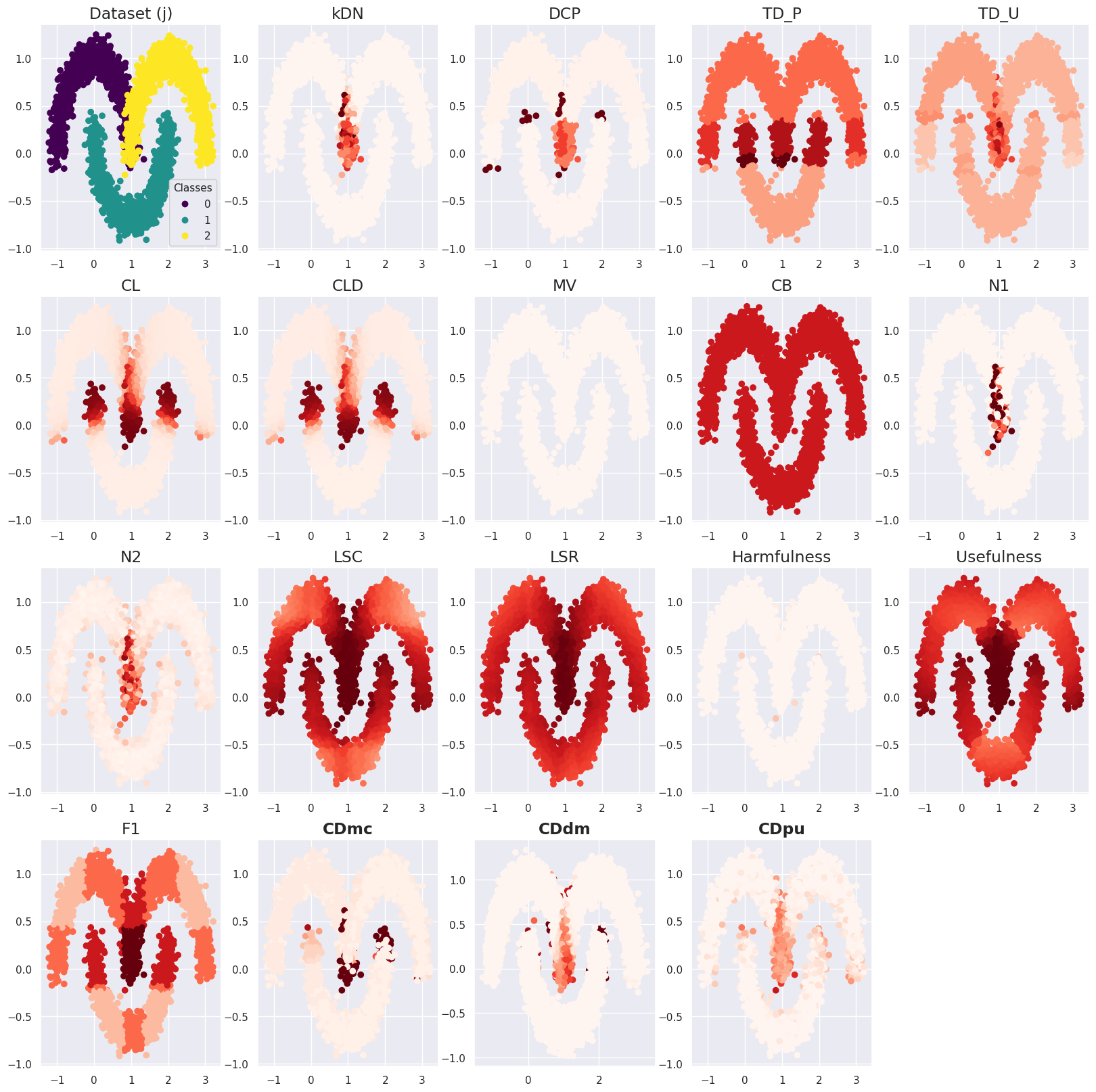


**Supplementary Figure S10.** Case difficulty of the dataset (j) from the existing metrics and the proposed metrics. CDdm results were calculated using four times more samples than CDmc and CDpu because more training data were required to train two models. Case difficulty ranges from 0 to 1, with an easy case being colored light red and a hard case being colored dark red.


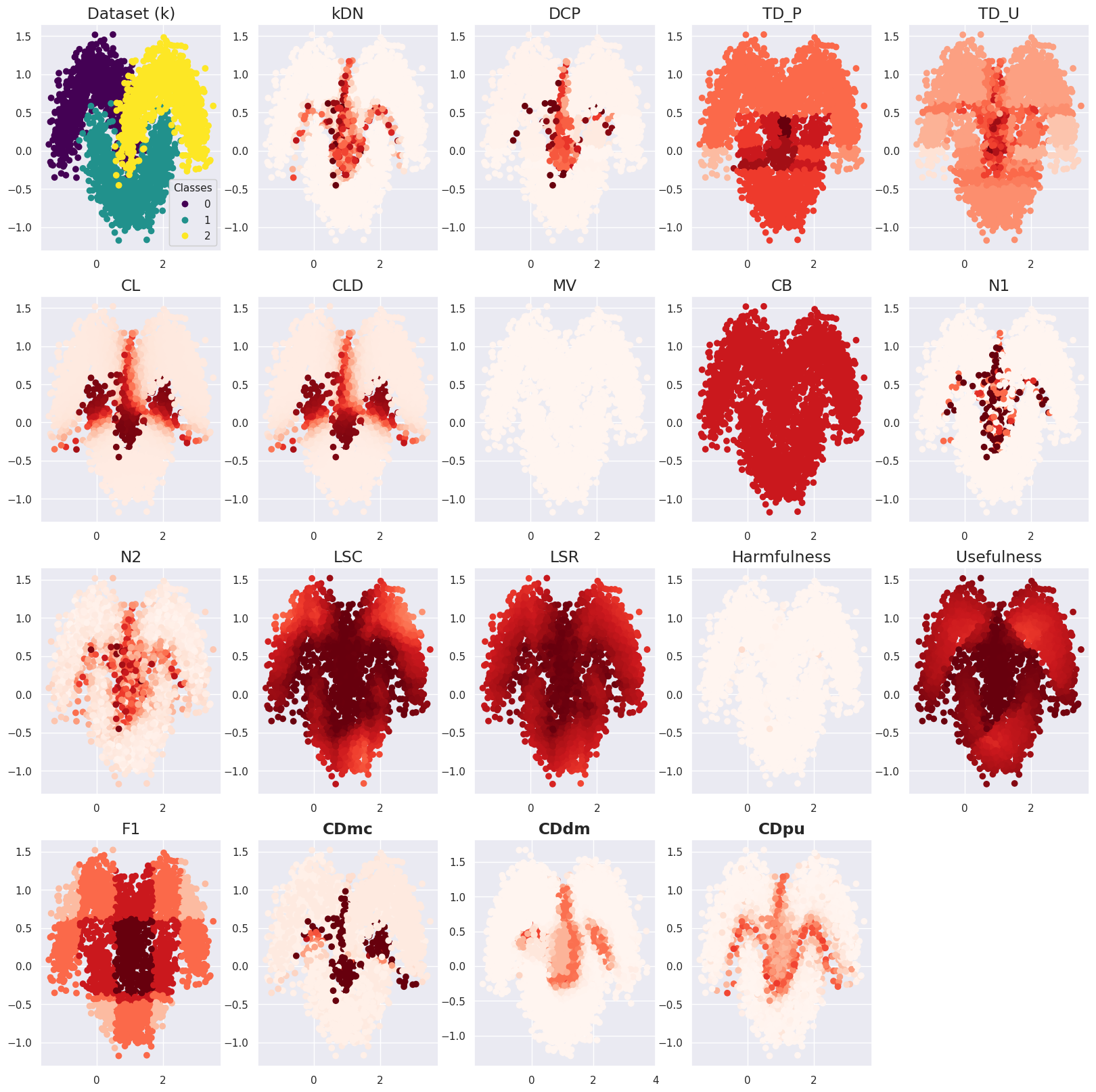


**Supplementary Figure S11.** Case difficulty of the dataset (k) from the existing metrics and the proposed metrics. CDdm results were calculated using four times more samples than CDmc and CDpu because more training data were required to train two models. Case difficulty ranges from 0 to 1, with an easy case being colored light red and a hard case being colored dark red.


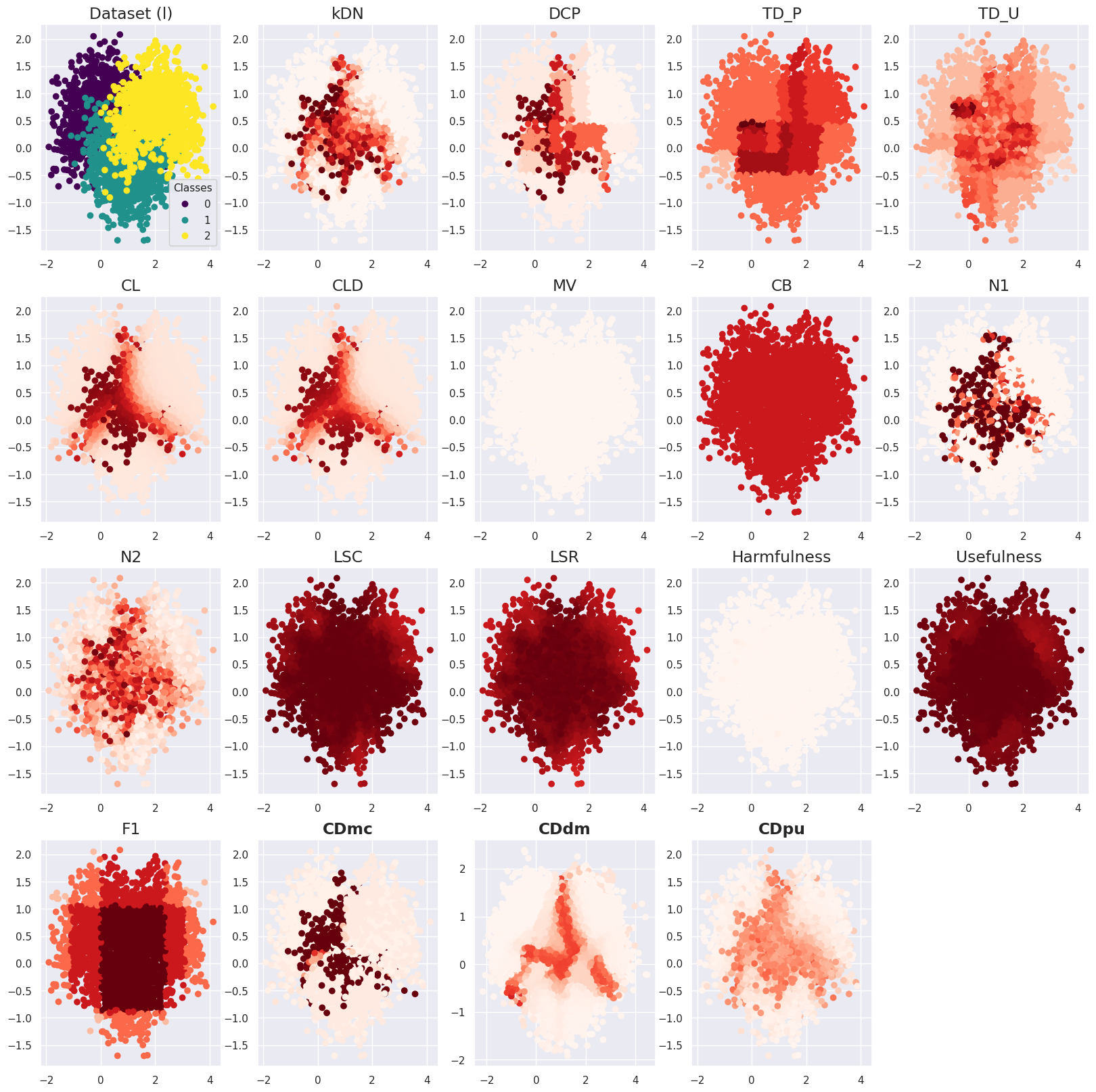


**Supplementary Figure S12.** Case difficulty of the dataset (l) from the existing metrics and the proposed metrics. CDdm results were calculated using four times more samples than CDmc and CDpu because more training data were required to train two models. Case difficulty ranges from 0 to 1, with an easy case being colored light red and a hard case being colored dark red.


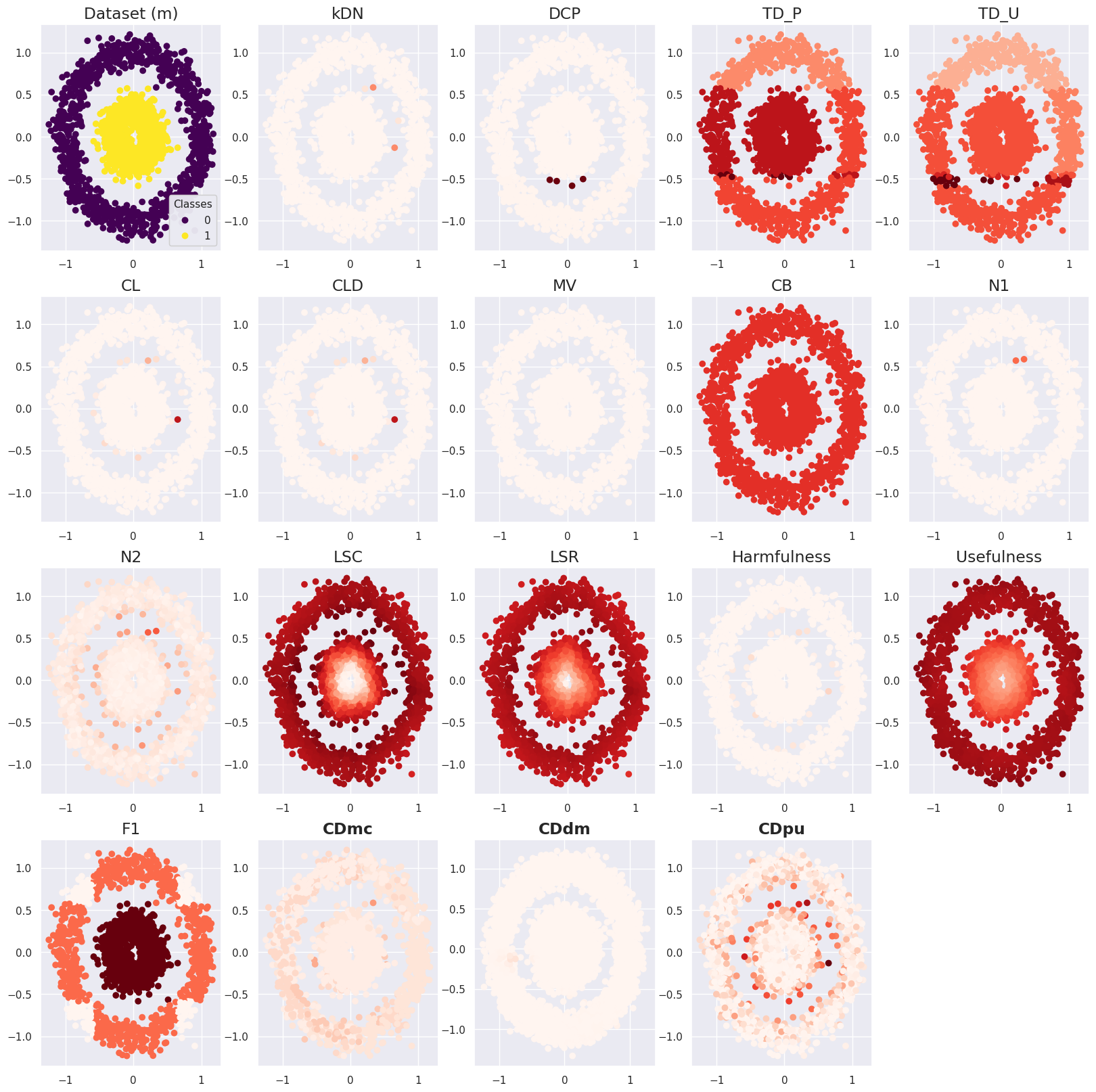


**Supplementary Figure S13.** Case difficulty of the dataset (m) from the existing metrics and the proposed metrics. CDdm results were calculated using four times more samples than CDmc and CDpu because more training data were required to train two models. Case difficulty ranges from 0 to 1, with an easy case being colored light red and a hard case being colored dark red.


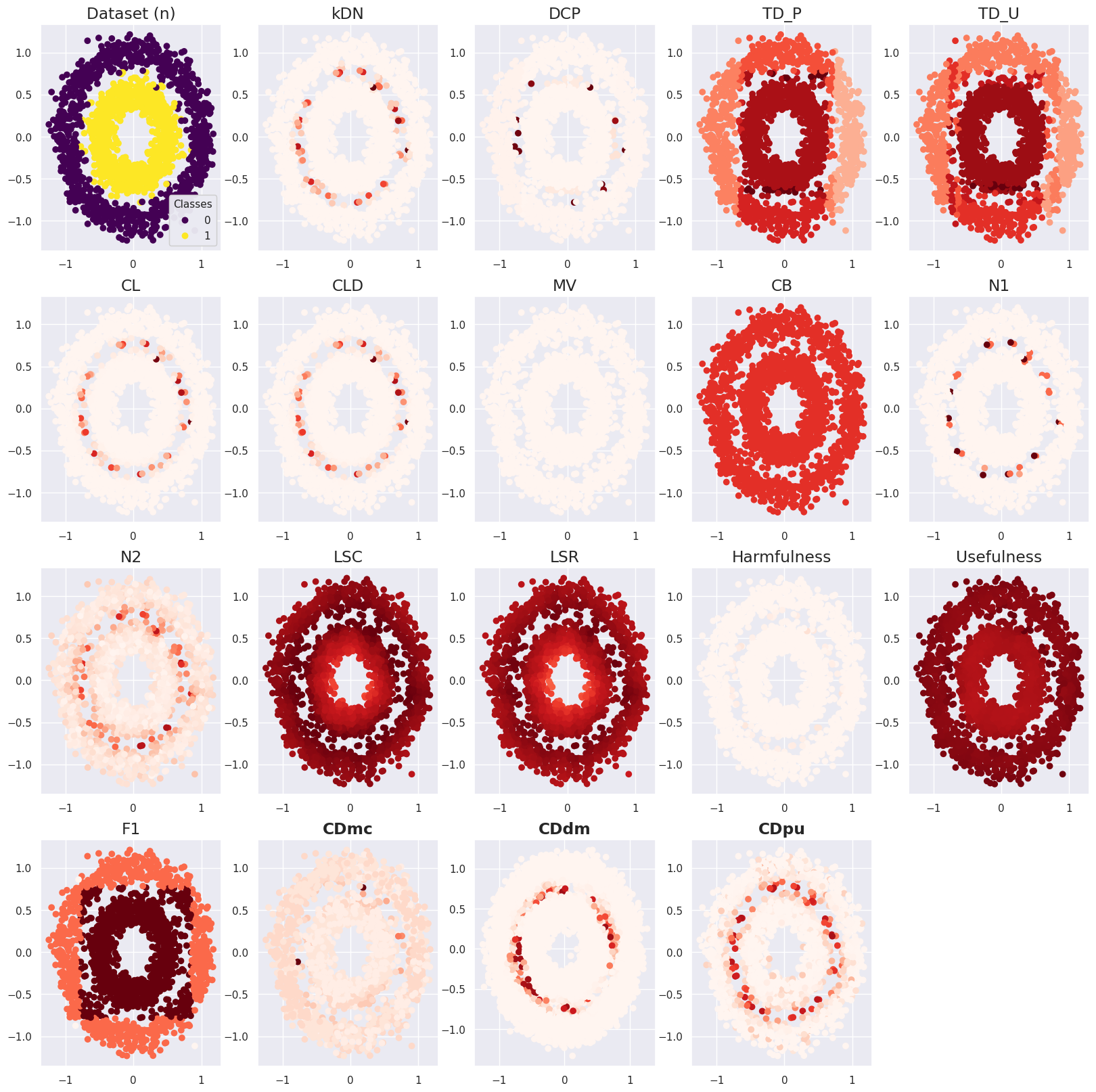


**Supplementary Figure S14.** Case difficulty of the dataset (n) from the existing metrics and the proposed metrics. CDdm results were calculated using four times more samples than CDmc and CDpu because more training data were required to train two models. Case difficulty ranges from 0 to 1, with an easy case being colored light red and a hard case being colored dark red.


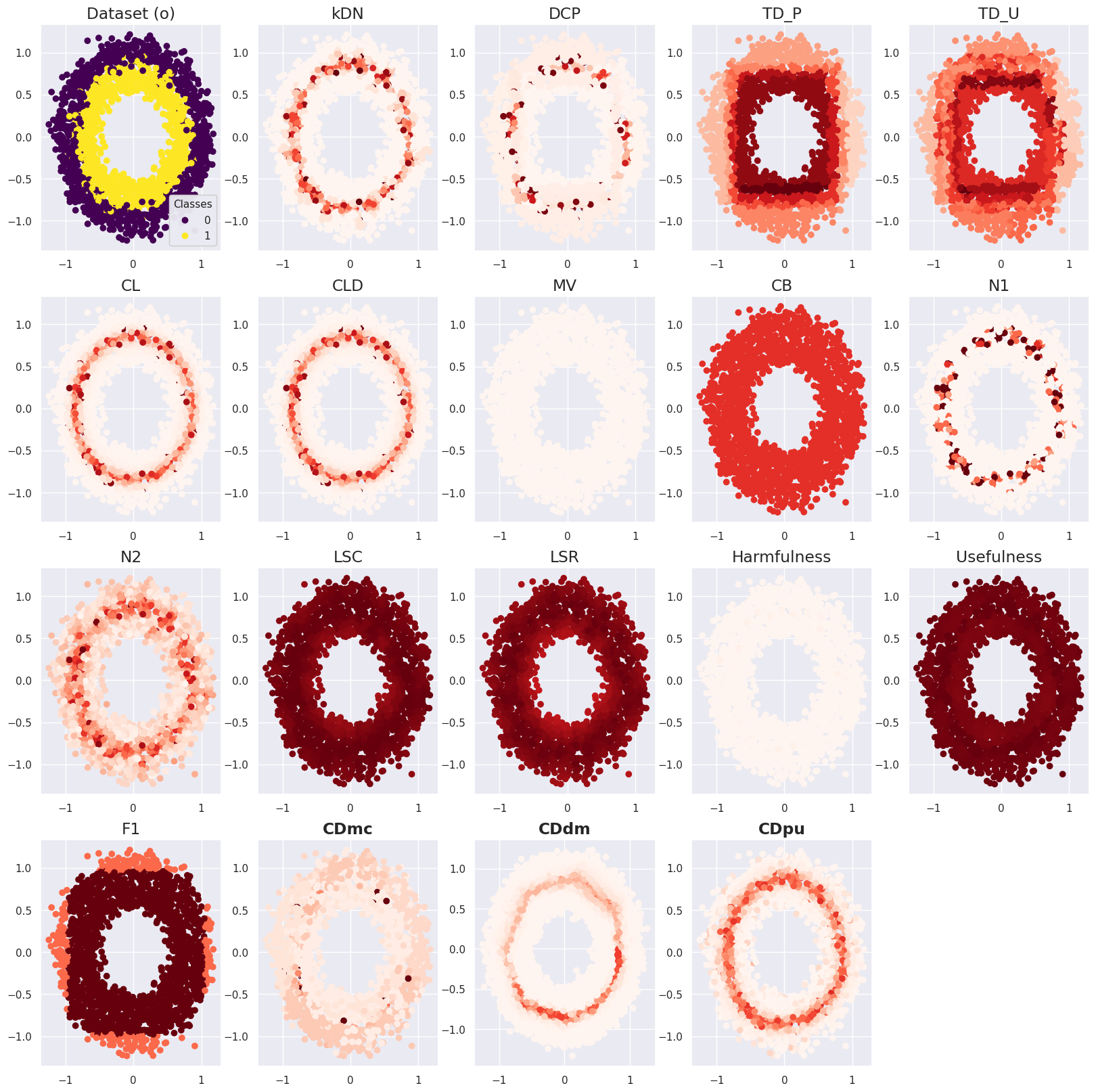


**Supplementary Figure S15.** Case difficulty of the dataset (o) from the existing metrics and the proposed metrics. CDdm results were calculated using four times more samples than CDmc and CDpu because more training data were required to train two models. Case difficulty ranges from 0 to 1, with an easy case being colored light red and a hard case being colored dark red.


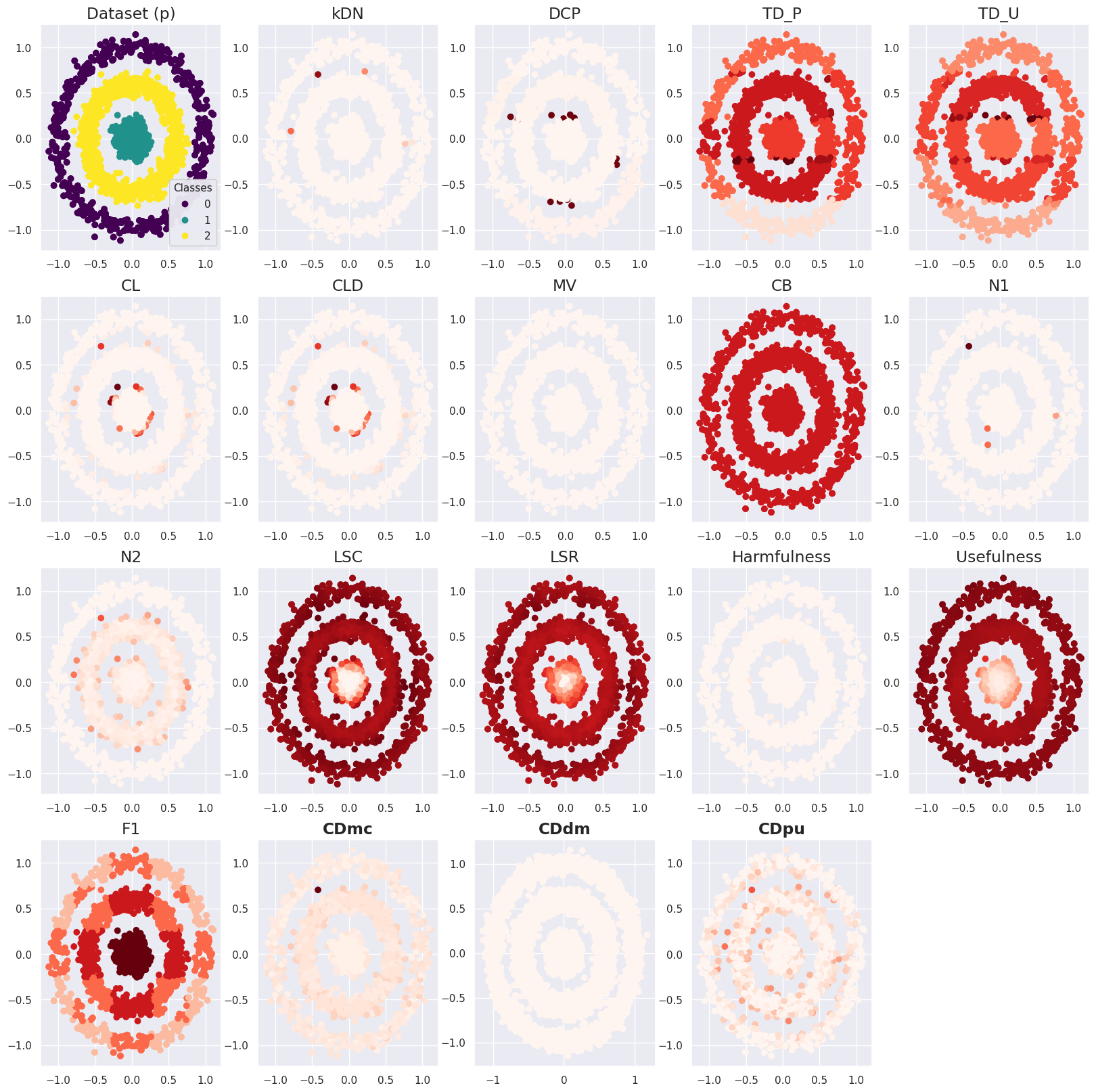


**Supplementary Figure S16.** Case difficulty of the dataset (p) from the existing metrics and the proposed metrics. CDdm results were calculated using four times more samples than CDmc and CDpu because more training data were required to train two models. Case difficulty ranges from 0 to 1, with an easy case being colored light red and a hard case being colored dark red.


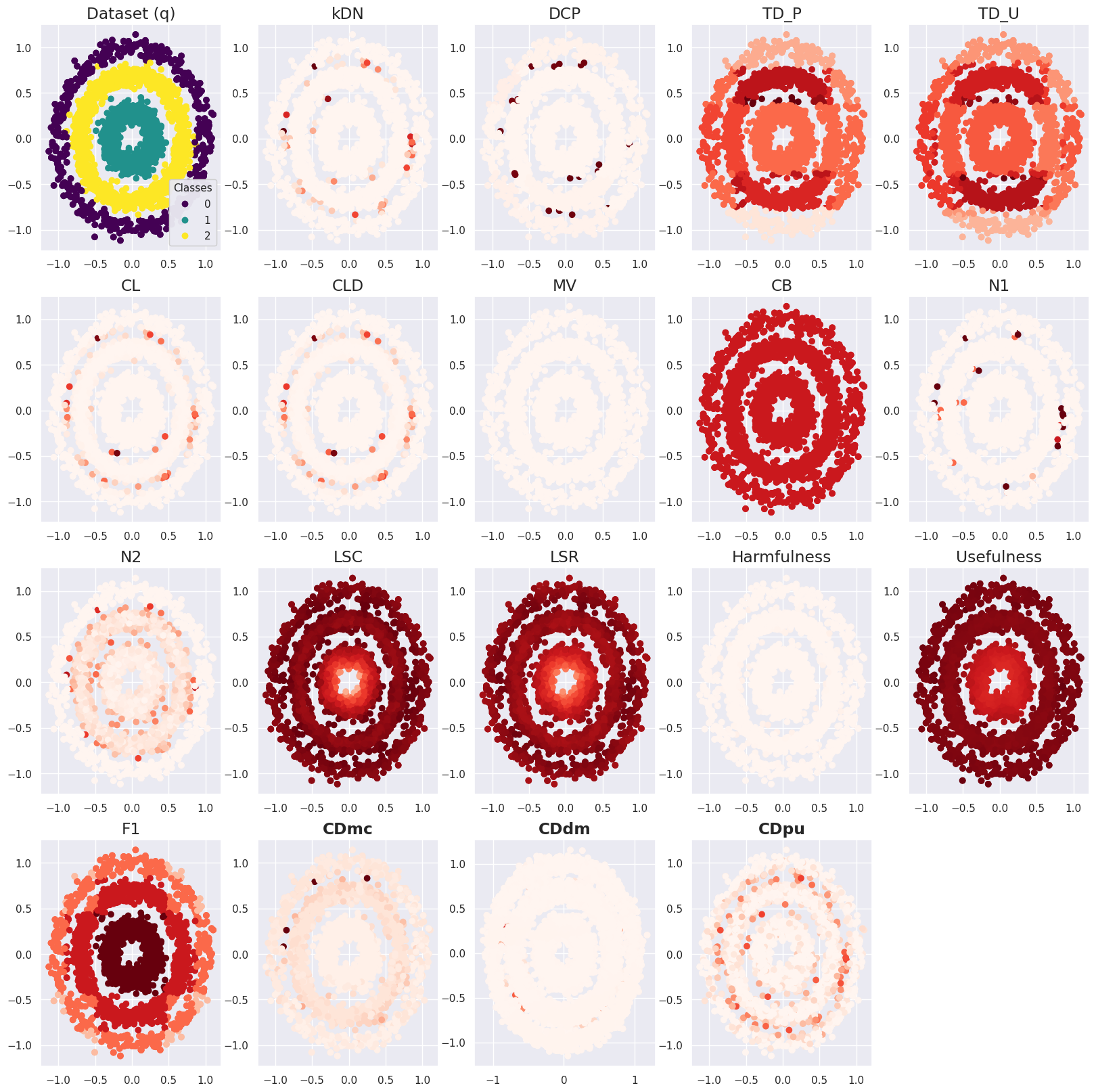


**Supplementary Figure S17.** Case difficulty of the dataset (q) from the existing metrics and the proposed metrics. CDdm results were calculated using four times more samples than CDmc and CDpu because more training data were required to train two models. Case difficulty ranges from 0 to 1, with an easy case being colored light red and a hard case being colored dark red.


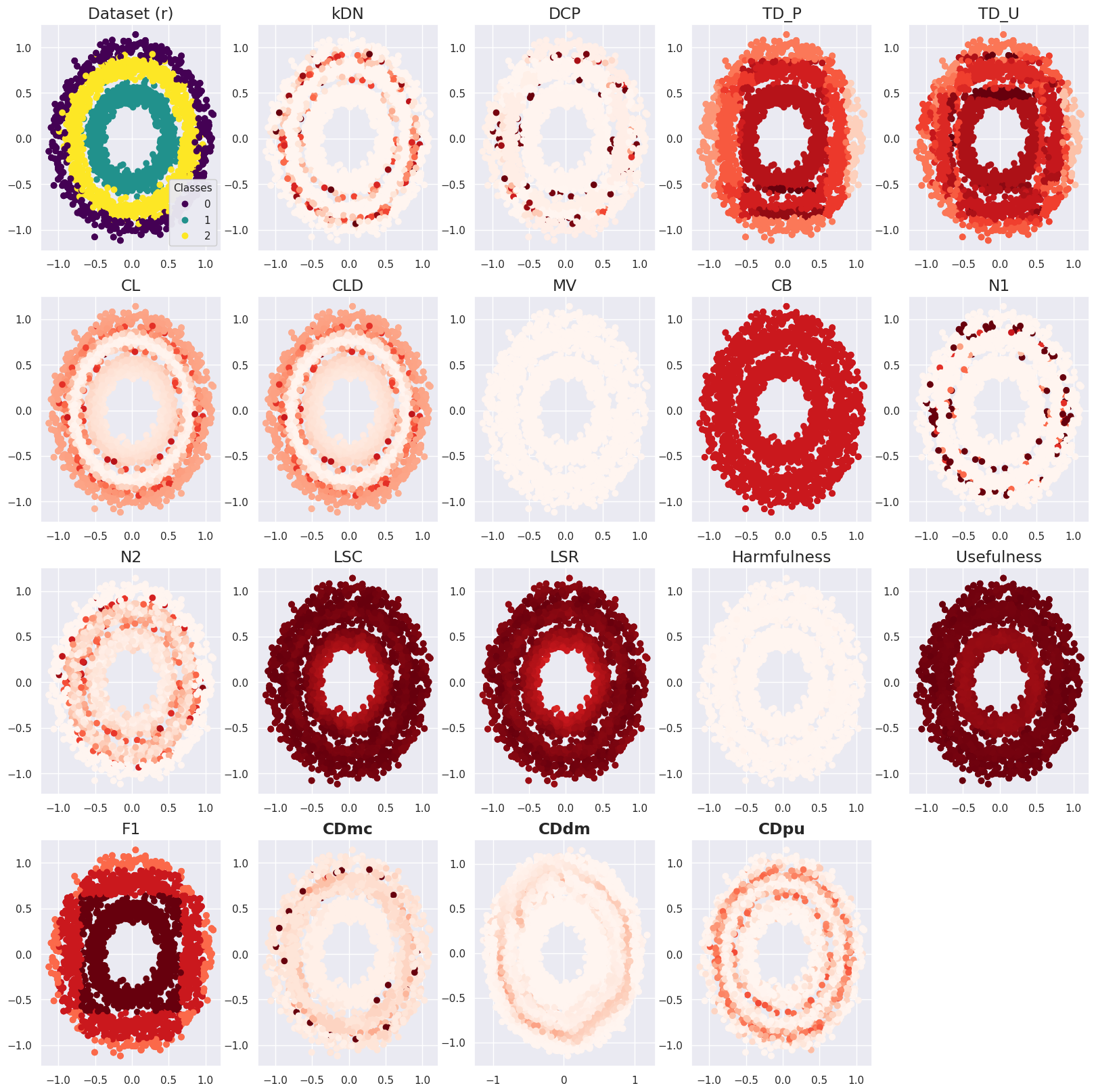


**Supplementary Figure S18.** Case difficulty of the dataset (r) from the existing metrics and the proposed metrics. CDdm results were calculated using four times more samples than CDmc and CDpu because more training data were required to train two models. Case difficulty ranges from 0 to 1, with an easy case being colored light red and a hard case being colored dark red.


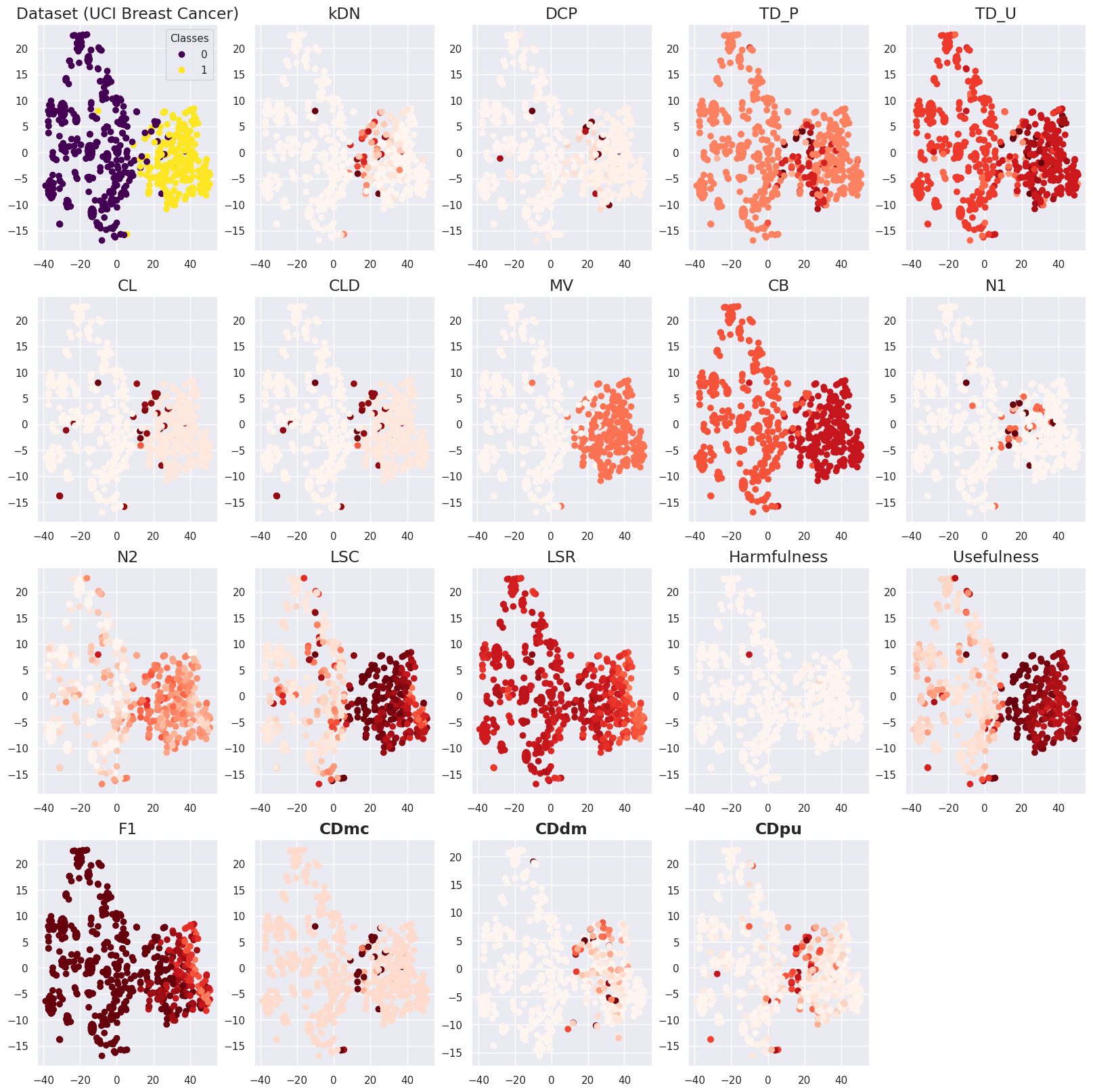


**Supplementary Figure S19.** Case difficulty of the UCI breast cancer data from the existing metrics and the proposed metrics. The t-SNE was used to visualize the UCI breast cancer data and the number of principal components was set to 2 to plot the results in a two-dimensional space. The case difficulty scale ranges from 0 to 1, with the lightest color indicating a low case difficulty, and the darkest color indicating a high case difficulty.


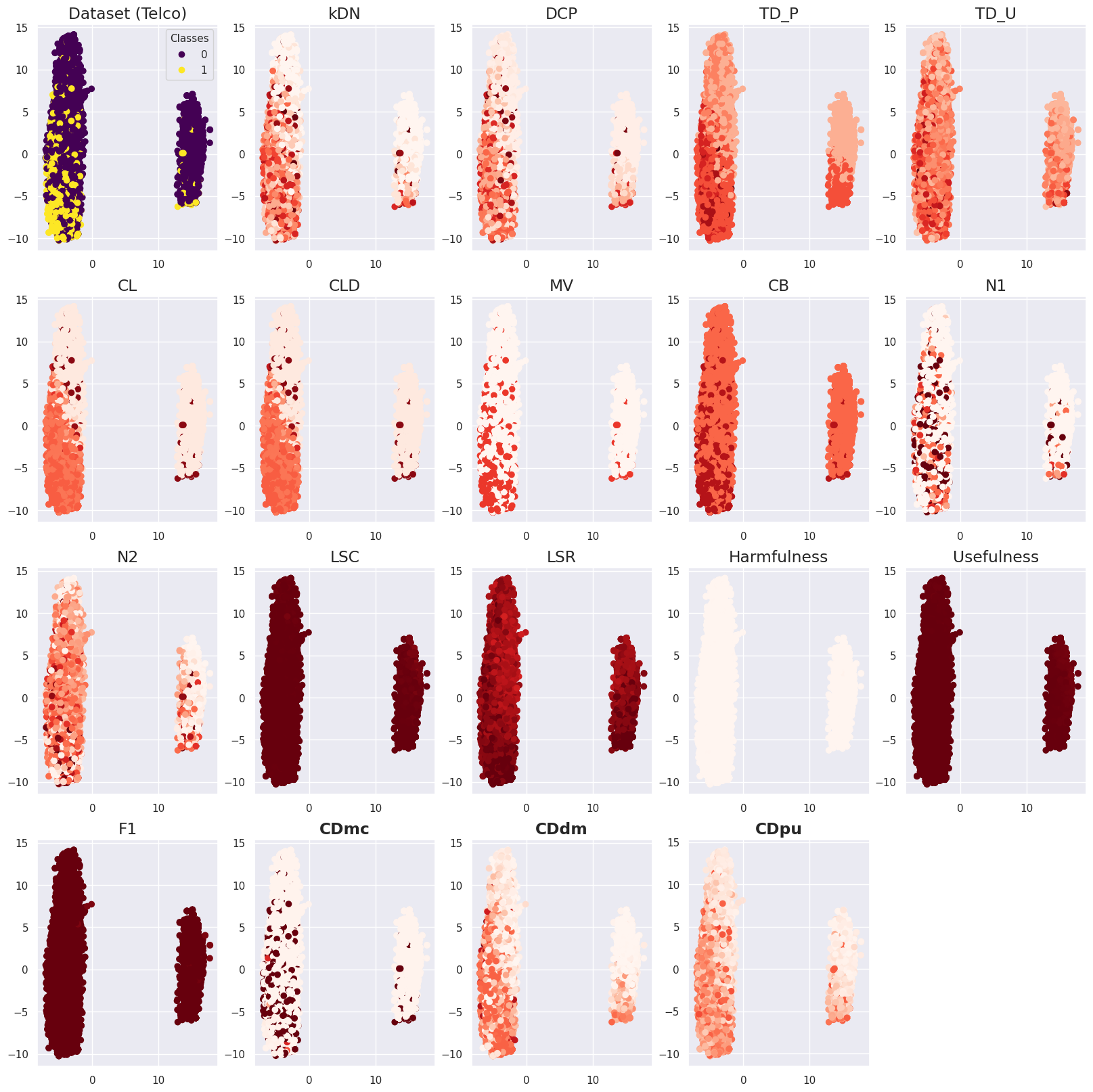


**Supplementary Figure S20.** Case difficulty of the Telco data from the existing metrics and the proposed metrics. The FAMD was used to visualize the Telco data and the number of principal components was set to 2 to plot the results in a two-dimensional space. The case difficulty scale ranges from 0 to 1, with the lightest color indicating a low case difficulty, and the darkest color indicating a high case difficulty.


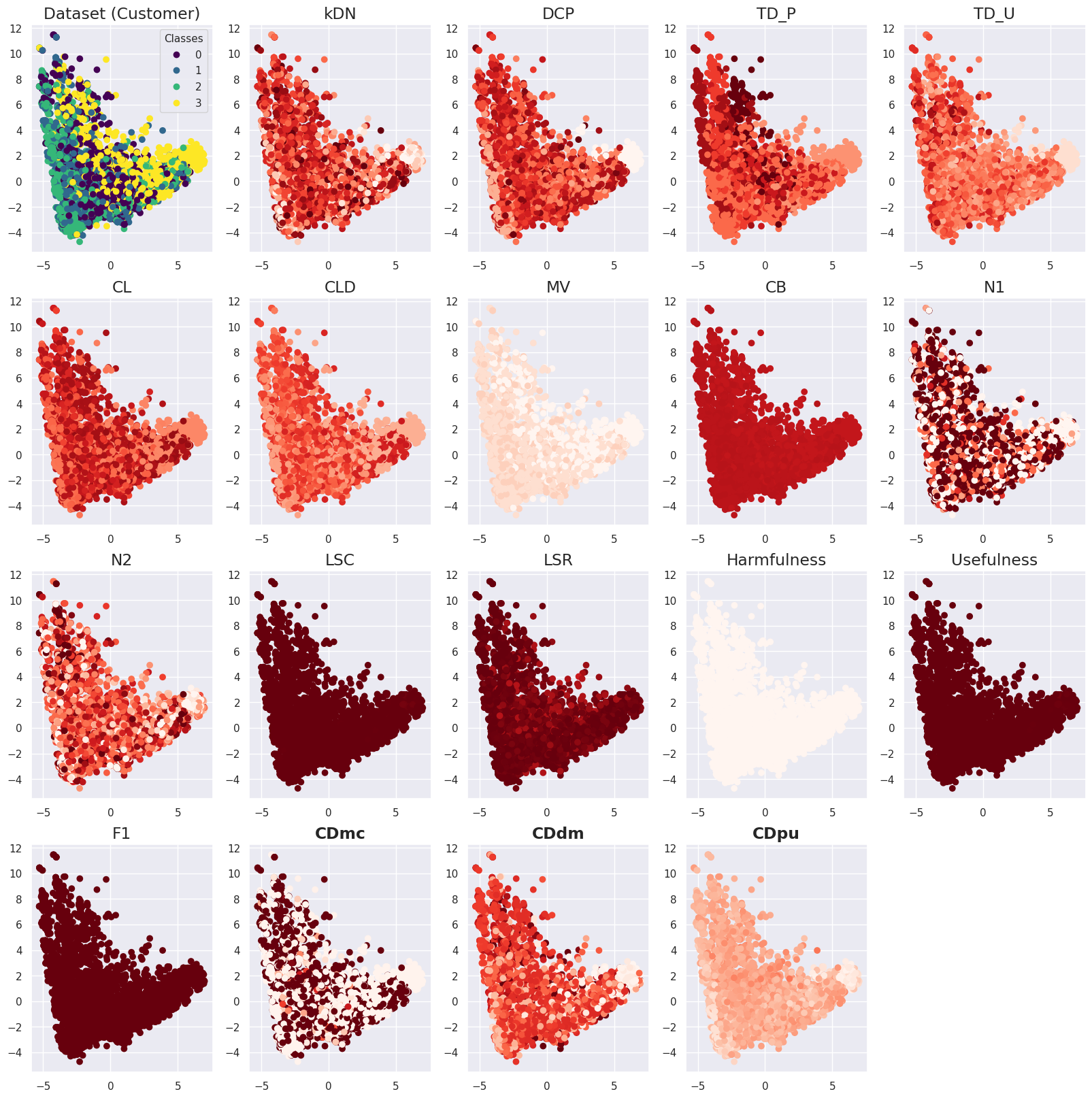


**Supplementary Figure S21.** Case difficulty of the Customer data from the existing metrics and the proposed metrics. The FAMD was used to visualize the Customer data and the number of principal components was set to 2 to plot the results in a two-dimensional space. The case difficulty scale ranges from 0 to 1, with the lightest color indicating a low case difficulty, and the darkest color indicating a high case difficulty.
